# Psychological “effects” of digital technology: a meta-analysis

**DOI:** 10.3389/fpsyg.2025.1560516

**Published:** 2025-10-03

**Authors:** Daniela-Elena Lițan

**Affiliations:** Department of Psychology, West University of Timișoara, Timișoara, Romania

**Keywords:** digital transformation, digital revolution, digital era, mental health, meta-analysis

## Abstract

**Introduction:**

The digital “revolution” brings along consequences at the individual level, consequences in terms of mental health, both positive and negative. Therefore, the purpose of the meta-analysis presented in this work is to investigate, in the adult population, the associated factors (psychological distress, anxiety, depression, stress, burnout, loneliness and social isolation, insomnia, and psychological well-being (PWB)) by means of digital technology represented by Artificial Intelligence (AI), remote work (RW), smartphone, social media (SM), and smart technologies used in tourism (STT).

**Methods:**

The meta-analysis was performed between June 2020–June 2024, and the protocol was registered in the PROSPERO database (CRD42024560285). Forty-seven papers involving a total of 36,100 participants were included in the meta-analysis. Standard meta-analytic procedures were applied, and correlation coefficients (r) were used as measures of effect size.

**Results:**

The highest positive value of the effect was obtained for the association between PWB and the use of the digital environment (AI, RW, and STT) *r* = 0.435, and the highest negative effect value was obtained for the association between burnout and the use of the digital environment (AI and RW) *r* = −0.478. The moderation analysis further clarified the role of contextual variables.

**Discussion/Conclusion:**

This meta-analysis highlights that digital technologies have both positive and negative effects on adult mental health, reflecting the complex impact of the digital environment.

**Systematic review registration:**

https://www.crd.york.ac.uk/PROSPERO/search, CRD42024560285.

## 1 Introduction

Digital environments are virtual places accessible through Internet connections. The most common forms of digital media are accessible through various devices, such as computers, consoles, smartphones, large SM platforms, and virtual experiences using headphones or other accessories (Forrest and Wexler, 2023). From a theoretical point of view, the digitalization phenomenon, as previously mentioned, does not seem to be very intrusive from a psychological point of view at first glance; however, through a closer analysis, we can notice how the elements become increasingly complex through technical ramifications, human interaction, and implicit psychological consequences. In the current specialized literature, the following digital entities are preferentially analyzed in relation to the most common psychological consequences, as shown in the meta-analysis below:

- *Smartphones*: Although smartphones can be used for many practical purposes, their many features increase the risk of overuse, a key element in addictive behavior (Aarestad et al., 2023).
- *SM*: Currently, social networks are used intensively by users to connect, follow news, and share information (Jiang and Ngien, 2020).
- *AI*: AI is defined as a broad set of computer-aided systems that integrate complex mathematical algorithms and effectively facilitate problem-solving and decision-making (Akerkar, 2019). Some common examples of such IT systems in today’s world are personal digital assistants (e.g., Apple’s Siri), chatbots, and voice-controlled navigation systems (Lteif and Valenzuela, 2022).
- *RW*: RW offers many new aspects to professional life, both positive and negative, such as working in virtual teams and mobile work. However, it also blurs the line between free time and working hours and creates the expectation of constant availability, the frequent need to adapt to digital changes, and the need to develop skills to use new digital tools (Bordi et al., 2018; Köffer, 2015); these elements generate stress, discomfort, and anxiety in employees (Azzahra et al., 2022). In other words, in addition to the countless undeniable advantages brought about by technology in all aspects of our lives, its massive and constant use, especially for performing work tasks, generates a series of “adverse effects” (Azzahra et al., 2022), such as technostress, increased workload, anxiety, burnout, fatigue, and isolation.

In addition, constant connectivity can cause work–family conflicts and/or negatively affect other aspects of an individual’s life (Ferguson et al., 2016). Moreover, Tarafdar et al. (2014) reported that the use of technology generates negative attitudes and emotions. Studies examining the increased prevalence of RW have also determined that individuals may face several adversities, including social withdrawal and decreased self-esteem (Tavares, 2017). All these factors can cause or worsen mental health problems (Teepe et al., 2023).

Undoubtedly, digitalization is not only used for professional purposes. Social networks represent a source of uncertainty and fear regarding mental health.

On the other hand, referring to AI, Salah et al. (2024) conducted a study in the online environment on 732 participants, showing that the perception of users concerning AI, represented by ChatGPT, as an unbiased and objective tool, is associated with increased PWB.

If, in the specialized literature, we find a series of recent meta-analyses (Ivie et al., 2020; Liu et al., 2022; Zhang et al., 2021) dedicated either to a certain type of digital environment (for example, smartphone, SM, etc.), or to a certain symptom identified in relation to the digital environment (for example, anxiety, depression, etc.), where the analyzed population mainly comprises teenagers, we might be able to change the paradigm. A meta-analysis of the entire digital environment for the time period June 2020–June 2024, dedicated to adults, considering the manifestation of symptoms including psychological distress, anxiety, depression, stress, burnout, loneliness and social isolation, insomnia, and PWB in relation to the entire digital environment, as it is currently known, can help us achieve it.

Considering all the aforementioned elements, we intend to answer the following question: What psychological factors are associated with the current “digital revolution”? We also investigated the potential moderators of the relationship between digitalization and the other variables.

## 2 Methodology

### 2.1 Searching strategy

The meta-analysis was performed following the PRISMA (Preferred Reporting Items for Systematic Reviews and Meta-Analyses) reporting guidelines (Liberati et al., 2009; Moher, 2009). The protocol was pre-registered in the PROSPERO database (The International Prospective Register of Systematic Reviews), receiving the registration code CRD42024560285 (the protocol can be accessed at the following address: https://www.crd.york.ac.uk/prospero/).

The Web of Science, MEDLINE, ProQuest, PsycINFO, Scopus, Elsevier, and PubMed electronic databases were systematically searched. Both published and unpublished materials (e.g., PhD theses and dissertations) written in English, dating from January 1, 2020, to June 20, 2024, were considered. The following phrases were used to conduct the search: (“digital transformation” OR “artificial intelligence” OR “internet of things” OR “IoT” OR smart* OR “remote work” OR “work from home” OR “distance work” OR “online business” OR “online school” OR “chatgpt” OR “chatbot” OR “voice-activated technolog*” OR “digital era”) AND (“mental health” OR “psychological health” OR “psychological well-being” OR “emotional well-being” OR “mental wellness” OR “depression” OR “anxiety” OR “sleep disturbance” OR “burnout” OR “distress” OR “social isolation” OR “social withdrawal” OR “worr*”). The search was performed using the titles, abstracts, and keywords in all the electronic databases mentioned above. These were independently evaluated by two reviewers. The reviewers resolved disagreements through discussion after independently coding the characteristics of the disputed studies.

**Figure 1:**
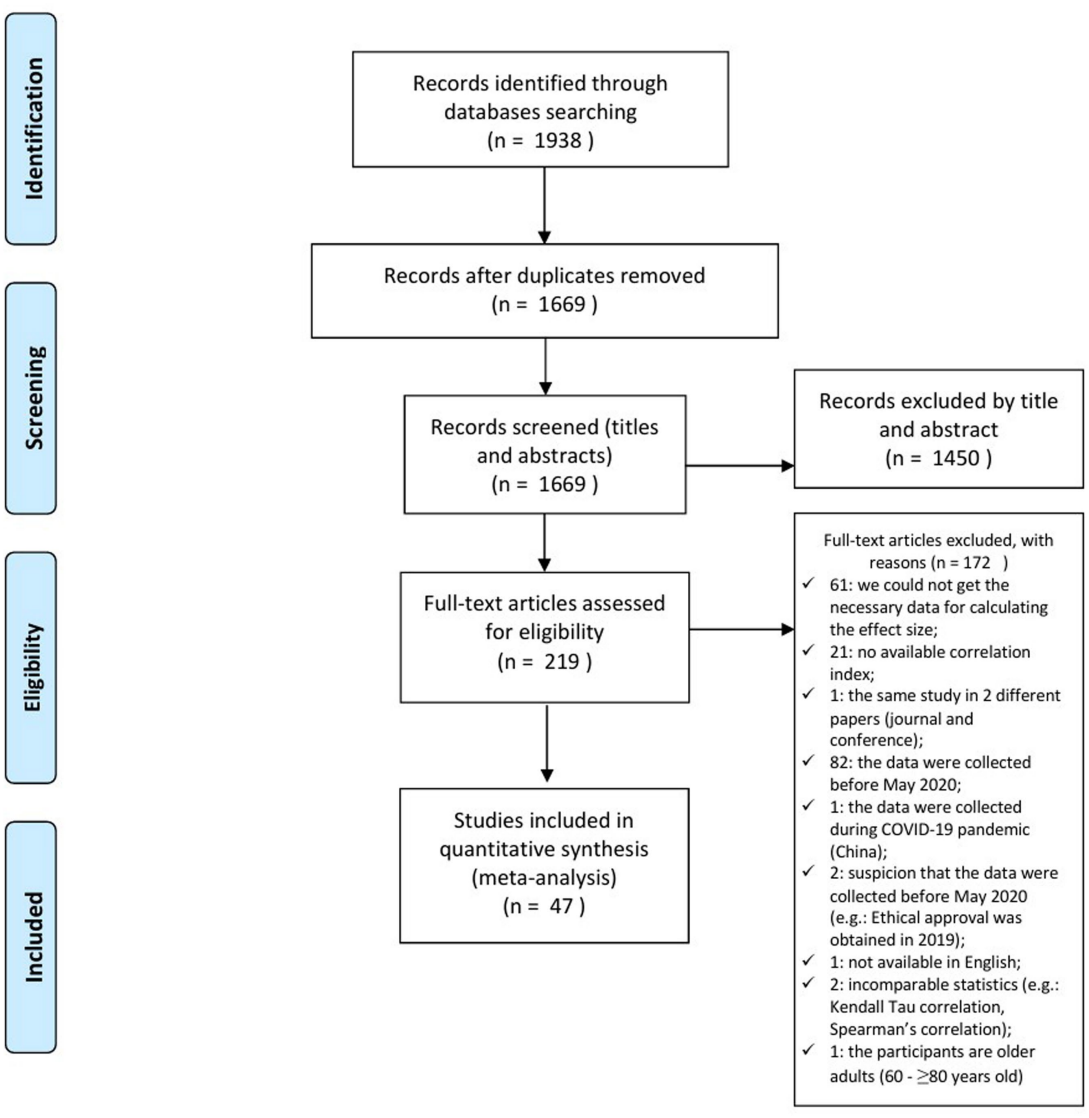
PRISMA flow diagram that illustrates the study selection process. *n* = number of evaluated studies.

Using this strategy, we obtained 1938 records (citations). Following the removal of duplicate records and those evaluated based on the title and abstract, 219 records with fully inspected content were obtained. After a full content analysis of the 219 studies, 47 papers with 77 independent studies were included in the meta-analysis. The entire process is illustrated in the PRISMA diagram in Figure 1.

### 2.2 Inclusion and exclusion criteria

We included observational studies (prospective, cohort type, or longitudinal design) that provided quantitative data on *psychological factors* (psychological distress, anxiety, depression, stress, burnout, loneliness and social isolation, insomnia, and PWB) associated with *digitalization* (smartphone, RW, AI, SM) in the adult population, regardless of social background, education, or country of origin, but without belonging to specific groups (e.g., veterans, military, people with disabilities, and specific physical or mental health problems).

In addition, to minimize potential bias caused by exceptional contexts, we excluded studies whose samples were collected during periods of pandemics (e.g., January–May 2020, the initial COVID-19 outbreak) or wars (e.g., studies conducted on the Ukrainian population from February 2022 onward). For studies that did not clearly indicate the period of sample generation, the timeframe was estimated using available metadata (e.g., received date, accepted date, published date, or ethical approval date), and those falling within restricted periods were also excluded. Furthermore, to ensure methodological consistency and comparability of effect sizes, only studies reporting Pearson’s correlation coefficients were included. Studies using alternative correlation measures (e.g., Spearman’s rho, Kendall’s tau) were excluded because they rely on different assumptions and scales, which could bias the aggregation of results in meta-analysis.

Studies that did not have sufficient quantitative information to calculate the effect size or that were not published in English were excluded. Papers featuring applications specifically developed to treat symptoms and pathologies or experimental applications developed for various devices (including mobile devices) were also removed.^1^

### 2.3 Data extraction and coding

The extracted characteristics of the included studies are presented in Table 1, which contains identifying information about each manuscript (e.g., authors and year of publication) and data on the study methods section (e.g., sample size, type of design, average age, and demographic information).

**Table 1:** Characteristics of included studies in the meta-analysis.

Study name	Study sample size	Gender ratio(% female)	Sample mean age	Demographics	Study design	Publication status	Digital environment	Digital environment use—indicator (psychological concept)	Variables associated with digitalization (symptoms) analyzed in this study	Study quality
Ostic et al. (2021)	940	76.49%	NS	Mexico students	Cross-sectional	Published	Smartphone	Addiction	Social isolation; PWB	2
El-Zoghby et al. (2024)	1,435	51.40%	21	Pakistani students	Cross-sectional	Published	Smartphone	Addiction	Psychological distress	1
Świątek et al. (2023)	210	85.00%	25	Polish citizens	Cross-sectional	Published	Smartphone	Problematic use	Burnout (SM Fatigue: cognitive, behavioral, emotional dimensions)	1
Ejaz et al. (2023)	427	49.60%	NS	Pakistani citizens	Cross-sectional	Published	Smartphone	Frequency	PWB; Anxiety - FoMO	1
Abdoli et al. (2023)	537	42.30%	25.52	Iranian students	Cross-sectional	Published	Smartphone	Nomophobia	Anxiety; Depression; Stress; Insomnia	1
Gonçalves and dos Santos (2022)	228	67.50%	32.32	Portuguese workers	Cross-sectional	Published	Smartphone	Addiction	Loneliness; Burnout	1
Sun et al. (2023)	577	53.70%	NS	Chinese corporate employees	Cross-sectional	Published	Smartphone	Addiction	Anxiety - Social Anxiety	2
Wickord and Quaiser-Pohl (2022)	399	78.19%	25.9	German students	Cross-sectional	Published	Smartphone	Problematic use	Depression; Anxiety	2
Servidio et al. (2022)	405	71.11%	22.11	Italian students	Cross-sectional	Published	Smartphone	Problematic use	Anxiety - FoMO	2
Lange and Kayser (2022)	5,163	53.10%	44	German workers	Cross-sectional	Published	RW (Work From Home)	RW experience	Anxiety - Job Anxiety	2
Xu et al. (2023)	321	53%	NS	Chinese workers	Cross-sectional	Published	AI	AI application (people positions/jobs replacement by AI - at work)	Burnout (Emotional exhaustion); Depression	1
Chen C, et al. (2023)	2,110	48.82%	NS	Chinese students	Cross-sectional	Published	Smartphone	Dependence	Burnout (Learning burnout)	1
Mazzei et al. (2023)	109	65%	NS	Italian workers	Cross-sectional	Published	RW (Hybrid work)	Affective commitment to the organization	Social isolation (Workplace social isolation); Stress (Technostress)	1
Al-Mamun et al. (2023)	585	54.70%	22.52	Bangladesh students	Cross-sectional	Published	Smartphone	Addiction	Depression; Insomnia	2
West et al. (2021)	94	67%	19.34	U. S. students	Cross-sectional	Published	Smartphone	Addiction	Depression	1
Peleg and Boniel-Nissim (2024)	431	65.20%	29.05	Israeli adults	Cross-sectional	Published	Smartphone	Phubbing	Anxiety - FoMO; Loneliness	2
Chang et al. (2024)	301	48.80%	NS	Chinese workers	Longitudinal	Published	AI	AI adoption intention	Anxiety - AI anxiety; PWB (Positive affect)	1
Teo et al. (2023)	53	NS	NS	Singaporean students	Cross-sectional	Published	Smartphone	Addiction	Depression; Stress (Perceived Stress)	1
Thatkar et al. (2021)	464	62.30%	NS	Indian students	Cross-sectional	Published	Smartphone	Addiction	Anxiety - Global interaction anxiousness (social interactional situations)	1
Zern (2023)	101	64.40%	37	U. S. corporate workers	Cross-sectional	Unpublished	RW (Work From Home)	Engagement	Burnout (Exhaustion)	1
Yang H, et al. (2022)	320	62.81%	20	Chinese students	Cross-sectional	Published	Smartphone	Addiction	Anxiety; Depression; Stress	2
Al Adwan et al. (2024)	300	27.30%	NS	Media workers in Egypt, France, and United Arab Emirates (UAE)	Cross-sectional	Published	AI	AI techniques (utilizing)	Anxiety - Professional future	1
Kim et al. (2024)	408	NS	NS	South Korean workers	Longitudinal	Published	AI	AI use (self-efcacy in AI use)	Anxiety - Job insecurity; Depression	1
Gani et al. (2023)	243	52.70%	NS	Bangladesh tourists	Cross-sectional	Published	STT	Automation (STT use)	PWB (Tourist PWB)	1
Presbitero and Teng-Calleja (2023)	345	65%	35	Country NS - call center agents	Longitudinal	Published	AI	Perceived AI	Anxiety - Job insecurity; Psychological distress	1
Kumar and Kumar (2024)	382	40%	32.28	Indian millennial tourists	Cross-sectional	Published	SM	Addiction	Anxiety - FoMO	1
Jin (2024)	321	60.40%	49	U. S. residents	Cross-sectional	Published	SM (Metaverse)	Metaverse adoption for virtual learning	Anxiety - Social phobia	2
Sommantico et al. (2023)	311	66.20%	23.5	Italian citizens	Cross-sectional	Published	SM	Addiction	Depression; Anxiety - FoMO	1
Yue et al. (2022)	573	56.37%	20.2	Chinese students	Cross-sectional	Published	Smartphone	Addiction	Loneliness	2
Liu et al. (2023)	5,909	53.80%	NS	Chinese students	Cross-sectional	Published	Smartphone	Addiction	Anxiety - Fear of negative evaluation	2
Salah et al. (2024)	732	53.41%	NS	Iraqi students	Cross-sectional	Published	AI (ChatGPT)	ChatGPT’s User Perceptions	PWB; Anxiety - Job Anxiety	1
Bermingham et al. (2021)	181	80.10%	21.45	U. S. students	Longitudinal	Published	Smartphone	Problematic use	Anxiety - Experiences of Close Relationships (Attachment anxiety); Loneliness - Capacity to Be Alone (Solitary)	1
Wang et al. (2024)	606	78.10%	NS	Chinese pre-service teachers	Cross-sectional	Published	AI (Generative AI)	Technological Pedagogical Content Knowledge	Anxiety - AI anxiety	1
Pourafshari et al. (2022)	2,569	93.10%	NS	Iranian citizens	Cross-sectional	Published	Smartphone	Addiction (overuse)	Anxiety; Depression; Stress	1
Wu et al. (2024)	2,993	65.70%	19.7	Tibetan students	Longitudinal	Published	Smartphone	Problematic use	Anxiety; Depression	1
Chaudhuri et al. (2022)	471	Asia: 32%; Europe: 29%	NS	Asia and Europ. citizens	Cross-sectional	Published	RW (Work From Anywhere)	Work from anywhere	PWB	1
Stevic and Matthes (2023)	840	55%	20.99	German citizens	Cross-sectional	Published	Smartphone	Co-use	Social isolation	1
Yang Z, et al. (2022)	207	72%	20.79	Chinese students	Cross-sectional	Published	Smartphone	Addiction	Depression; Stress; Anxiety - FoMO; Loneliness	2
Liu et al. (2024)	721	25.50%	19.4	Chinese students	Cross-sectional	Published	Smartphone	Problematic use	Anxiety - Social Anxiety	1
Hussain et al. (2024)	461	59.90%	24.15	online users and UK students	Cross-sectional	Published	Smartphone	Problematic use	Anxiety - FoMO	1
Brailovskaia and Margraf (2023)	185	77.30%	29.45	online users and German students	Longitudinal	Published	Smartphone	Problematic use	Depression	1
Parent et al. (2023)	403	76.60%	20.4	Canadian students	Cross-sectional	Published	Smartphone	Problematic use	Anxiety - Attachment Anxiety	1
Chen S, et al. (2023)	402	56.46%	22.48	Chinese students	Cross-sectional	Published	Smartphone	Problematic use	Loneliness	1
Geng et al. (2021)	355	83.10%	19.42	Chinese students	Cross-sectional	Published	Smartphone	Addiction	Anxiety; Depression	2
de Hesselle and Montag (2024)	86	100.00%	NS	German students (or good German language speakers students)	Longitudinal	Published	Smartphone	Problematic use	Loneliness	1
Tang et al. (2023)	166	47%	34.3	Taiwanese engineers	Cross-sectional	Published	AI (working with AI)	Interaction with AI (frequency)	Loneliness; Anxiety - Attachment Anxiety; Insomnia	1
Maftei and Măirean (2023)	720	74%	24.12	Romanian citizens	Cross-sectional	Published	Smartphone	Phubbing (perceived, experience)	Loneliness; Psychological distress	1

PWB, Psychological Well-Being; FoMO, Fear of Missing Out; RW, Remote Work; AI, Artificial Intelligence; STT, Smart tourism technologies; SM, Social Media.

For the general concept of anxiety, as found in the analyzed studies, studies that referred to the following subcategories were also included: FoMO (fear of missing out), social phobia, job insecurity, fear of negative evaluation, social anxiety,^2^ anxiety about working with AI, anxiety about the professional future, global interaction anxiety (social interaction situations), work anxiety, and attachment anxiety.

For the general concept of stress, as found in the analyzed studies, studies referring to the following subcategories were also included: technostress and perceived stress.

For the general concept of burnout, as found in the analyzed studies, studies referring to the following subcategories were also included: SM Fatigue (cognitive, behavioral, and emotional dimensions), emotional exhaustion, learning burnout, and exhaustion, taking into consideration that according to the APA Dictionary of Psychology, burnout is defined as “physical, emotional, or mental exhaustion accompanied by decreased motivation, decreased performance, and negative attitudes toward self and others.”

The concepts of loneliness and social isolation were grouped into the same category and subsequently analyzed together. Subcategories of social isolation at work and the ability to be alone (solitary) were also included.

For the general concept of PWB, as found in the analyzed studies, studies referring to the positive affect subcategory were also included. Positive affect is considered a part of PWB, as stated in The Mental Health Inventory (MHI-38; Pais-Ribeiro, 2001).

For transparency, all subscales (e.g., FoMO, technostress, emotional exhaustion) and their categorization into broader constructs (anxiety, stress, burnout, loneliness, PWB) are presented in Supplementary Table S1.

The remaining concepts, psychological distress, depression, and insomnia, did not require any coding or grouping.

The contribution of the following *potential moderators*, as coded below, was also analyzed:^3^

- The types of digital environment (digital entity) included SM (metaverse), AI, smartphone, RW, and STT.
- Digital environment use (psychological concept) included addiction, problematic use, AI use, frequency, AI adoption intention, AI user perceptions, nomophobia, phubbing, RW experience, job replacement by AI, interaction with AI (frequency), affective commitment to the organization, and co-use.
- Professional activities included students, employees, and other activities. In the other activity category, the categories of citizens and tourists, for which occupations were not mentioned in the corresponding study, were introduced.
- The geographic region moderator was divided into three categories, taking into account the characteristics of the included studies: Asia (Israel, China, Taiwan, Iran, Bangladesh, Singapore, South Korea, and Tibet), Europe (Portugal, Germany, Romania, and Italy), and North America (US and Mexico).
- Moderators: Country and average ages were drawn from the studies included in the analysis and did not require coding or clustering.

Given that psychological distress can be conceptualized as an “umbrella” term encompassing multiple common psychological conditions, ranging from sub-clinical symptoms to clinical diagnoses of depression, anxiety, stress, or post-traumatic stress disorder (Zhu et al., 2022), we also grouped these concepts into a single variable to have a clearer general picture of the psychological suffering generated by the digital “transformation.”

### 2.4 The quality of the studies

The NIH Quality Assessment Tool for Observational Cohort and Cross-Sectional Studies (National Institutes of Health, United tates) was used to assess the quality of the studies included in the meta-analysis. The questionnaire contained 14 questions regarding different aspects of the research and a general indicator of study quality: good (coded 1), average (coded 2), and poor (coded 3). The items assess the research question and objectives, sampling method, sample size justification, variables and outcome evaluation, and elements relevant to the meta-analysis (National Institute of Health, 2021).

Two independent reviewers examined the eligible studies. The reviewers resolved disagreements through discussion after independently coding the characteristics of the disputed study. All 47 studies were assessed as of good or medium quality. The reviewer agreement was optimal, with k = 0.685 (Cohen’s kappa; Cohen, 1960). The final evaluation of each study is shown in the study quality column in Table 1. The final quality assessment of the studies included in the meta-analysis concluded that 34 studies were of good quality and 13 were of medium quality.

### 2.5 Statistical analysis

The effect size assessment was performed using the correlation coefficient r (Pearson), which was extracted from each study included in the meta-analysis. The separate effect values were then combined for each of the factors (symptoms) assessed in the meta-analysis: psychological distress, anxiety, depression, stress, burnout, loneliness, social isolation, insomnia, and PWB. Both the random-effects and fixed-effects models were used to analyze the size of the effects, depending on the situation; if the result of the Q test (test for heterogeneity) was significant or the I^2^ value (total heterogeneity/total variability) was higher than 75%, the random-effects model was more appropriate; otherwise, the fixed-effects model was more appropriate (Huedo-Medina et al., 2006; Zhang et al., 2021).

The interpretation of the effect sizes was based on the recommendations of Cohen (1988): r values of 0.10 or less show a small effect size, values between 0.30 and 0.50 show medium effect sizes, and values of 0.50 or higher indicate large effect sizes.

Heterogeneity was assessed using the Q test and the percentage value of I^2^ (total heterogeneity/total variability). The significant values of the Q test indicate that the differences between the studies included in the meta-analysis are also due to factors other than sampling error (Măricuțoiu, 2020). If there is evidence of overall heterogeneity, constructing a Bajaut plot can highlight the studies that contribute to the overall heterogeneity and outcome (Quintana, 2015). To check and identify potential outliers that may have influenced the observed heterogeneity, a set of diagnostics derived from the standard linear regression available in the RStudio metaphor package was used (Viechtbauer and Cheung, 2010; Quintana, 2015). A *leave-one-out* analysis to evaluate the impact of each study in the meta-analysis on the overall effect size was also used (Borenstein et al., 2009). Publication biases were assessed by visual inspection of funnel plots and performing tests such as Egger’s regression and rank correlation (Quintana, 2015).

In the case of significant asymmetry (Egger’s test was significant), the Duval and Tweedie method was used (Duval and Tweedie, 2000). This method was used to estimate the true effect size if “missing” studies were published. This procedure adds missing studies to the funnel plot until symmetry is achieved (van Lissa, 2021). Subgroup analyses were also performed for the extracted categorical moderators (type of digital environment, use of digital environment, professional activity, country, and geographic region), as well as a meta-regression analysis for the continuous moderator average age. The entire analysis was performed using the RStudio software (version 2024.04.1 + 748) using the packages robumeta, metafor, dplyr (Quintana, 2015), and esc.

## 3 Results

### 3.1 Characteristics of studies included in the meta-analysis

The meta-analysis included 47 papers with 77 independent studies, whose samples were generated between July 2020 and October 2023, and included 36,100 participants, of whom 62.26% were women. The average age of the participants varied from 21.44 and 30.56 years. Most participants lived in Asia (28 samples) and Europe (14 samples), whereas others lived in North America (six samples) and Africa (one sample; Table 1). Thus, the studies included in this meta-analysis cover four continents (Asia, Europe, North America, and Africa), providing a broad, though uneven, geographic coverage of the adult population.

Regarding the types of studies (study designs) included in the meta-analysis, 65 studies were cross-sectional, and 12 were longitudinal. All the studies were published in specialized journals except for one, a doctoral thesis.

In all studies, both the measurement of digital environment use and the identification of symptoms (appearing in relation to the use of the digital environment) were based on specific self-administered scales.^4^

### 3.2 The association between digital environment (use) and other variables

Table 2 summarizes the results of the meta-analyses of the digital environment (usage) and the variables present in at least two studies. In the analysis below, the concept of the digital environment includes the following entities: AI, RW, STT (smartphones, Internet of Things [IoT]), smartphones, and SM.

**Table 2:** Summary of effect size coefficients.

Variable	k	N	r	95% CI	Q (df = k − 1)	I^2^(%)
Psychological distress *(measured in studies)*	3	2,500	0.396^***^	[0.214, 0.552]	36.54^***^	95.52%
Psychological distress *(grouped by anxiety, depression, stress, psychological distress measured in studies)*	51	29,896	0.287^***^	[0.224, 0.348]	1581.73^***^	97.84%
*Anxiety*	28	26,394	0.278^***^	[0.182, 0.369]	1302.85^***^	98.43%
*Depression*	14	9,337	0.275^***^	[0.161, 0.383]	204.76^***^	96.55%
*Stress*	6	3,795	0.296^***^	[0.166, 0.417]	22.15^**^	90.34%
Burnout^*^ Digital environment: Smartphone ^*^fixed effects model	3	2,548	0.278^***^	[0.242, 0.314]	1.17	0.0%
Burnout *Digital environment:* *AI,* *RW*	2	422	−0.478^*^	[−0.749, 0.070]	15.82^***^	93.68%
Loneliness and social isolation	12	4,883	0.191^***^	[0.075, 0.301]	111.14^***^	93.8%
Insomnia	3	1,288	0.217^*^	[0.043, 0.378]	24.47^***^	89.23%
PWB *Digital environment:* *AI,* *RW,* *STT*	4	1747	0.435^***^	[0.238, 0.598]	74.69^***^	95.25%

PWB, Psychological Well-Being; RW, Remote Work; AI, Artificial Intelligence; STT, Smart tourism technologies.

#### 3.2.1 The association between the general digital environment and psychological distress

For a broader picture of the psychological impact on the population following intense interaction with the digital environment, the correlation between the general digital environment and psychological distress was calculated in two forms:

- The measured values of *psychological distress* were kept as identified in the papers included in the meta-analysis, and the correlation coefficient obtained using the random-effects model of meta-analysis had a positive value of *r* = 0.396, 95% CI[0.214, 0.552], *p* < 0.001. Egger’s regression test was significant (Egger’s intercept = −0.0749, *p* < 0.0001), whereas the rank correlation test was insignificant (*p* = 0.3333). Given the results of Egger’s regression test (possible existence of publication bias), the Duval and Tweedie trim-and-fill procedures were also run, resulting in an estimate of zero missing studies. The next step was to perform a *leave-one-out* analysis, which did not reveal important variations in the overall effect size when studies were removed from the analysis individually.
- *Psychological distress as a grouping of the concepts of anxiety, depression, stress* (Derakhshanrad et al., 2021; Yang Z, et al., 2022) *and psychological distress measured in studies—*in this case, the correlation coefficient obtained using the random-effects meta-analysis model has a positive value of *r* = 0.287, 95% CI[0.224, 0.348], *p* < 0.001. After performing the statistical analysis to check and identify potential outliers (Bajaut diagram, diagnostics derived from the standard linear regression, *leave-one-out* analysis) and publication biases (funnel plot diagram, Egger’s regression test, rank correlation test) with no significant influence, the Duval and Tweedie trim-and-fill procedure showed the possibility of overestimating the initial result (*r* = 0.287, 95% CI[0.224, 0.348], *p* < 0.001) due to publication bias. However, for the “real” effect, when selective publication was controlled for, *r* = 0.217, 95% CI[0.150, 0.283], *p* < 0.001 rather than *r* = 0.287, 95% CI[0.224, 0.348], *p* < 0.001.^5^

#### 3.2.2 The association between the general digital environment and anxiety

**Figure 2:**
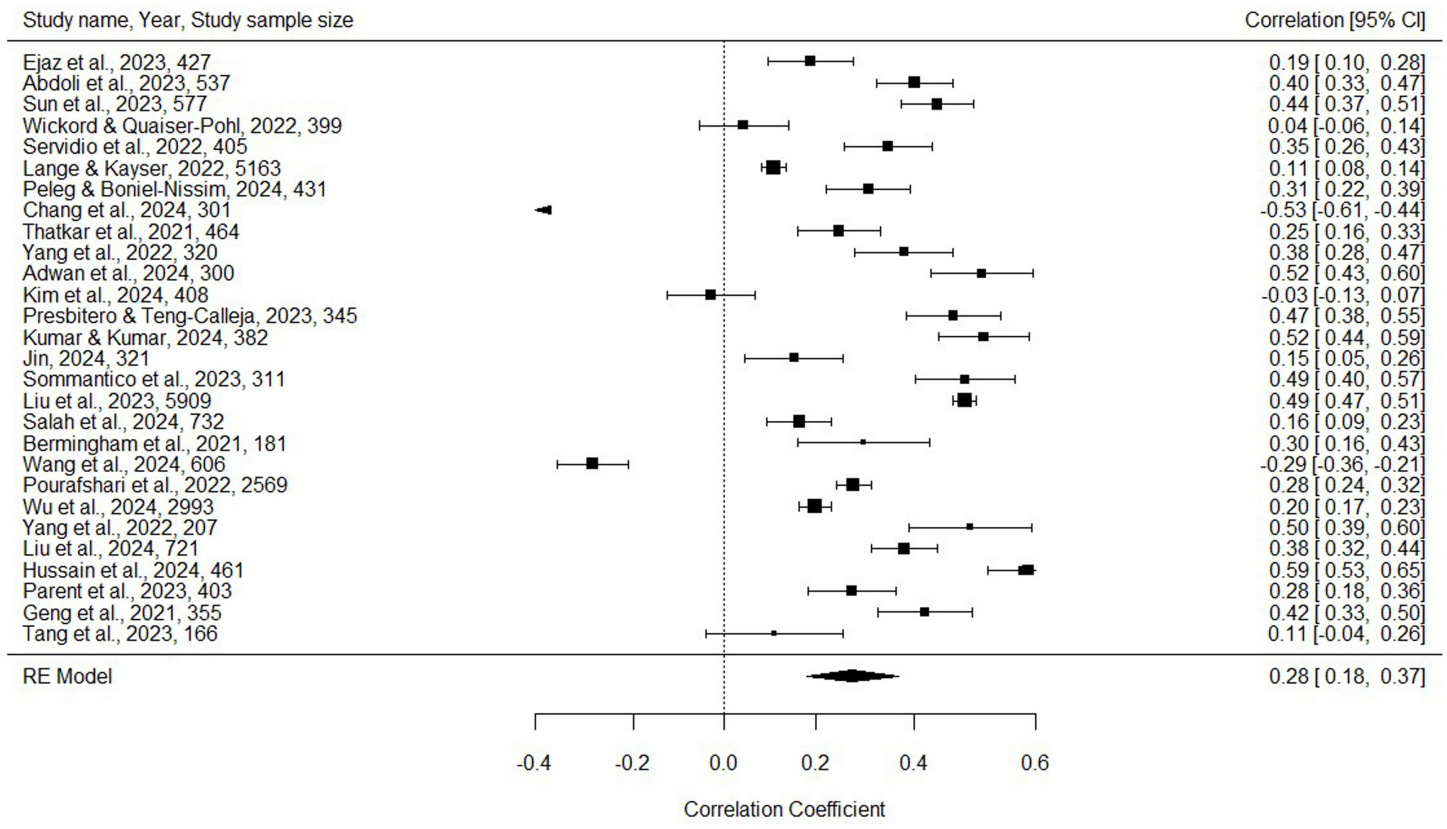
Forest plot diagram (anxiety and digital environment association).

The overall value of the obtained effect size, using the random-effects meta-analysis model regarding the association between the general digital environment (usage) and *anxiety*, is significant and positive (*r* = 0.278, 95% CI[0.182, 0.369], *p* < 0.001; see also Figure 2).

**Figure 3:**
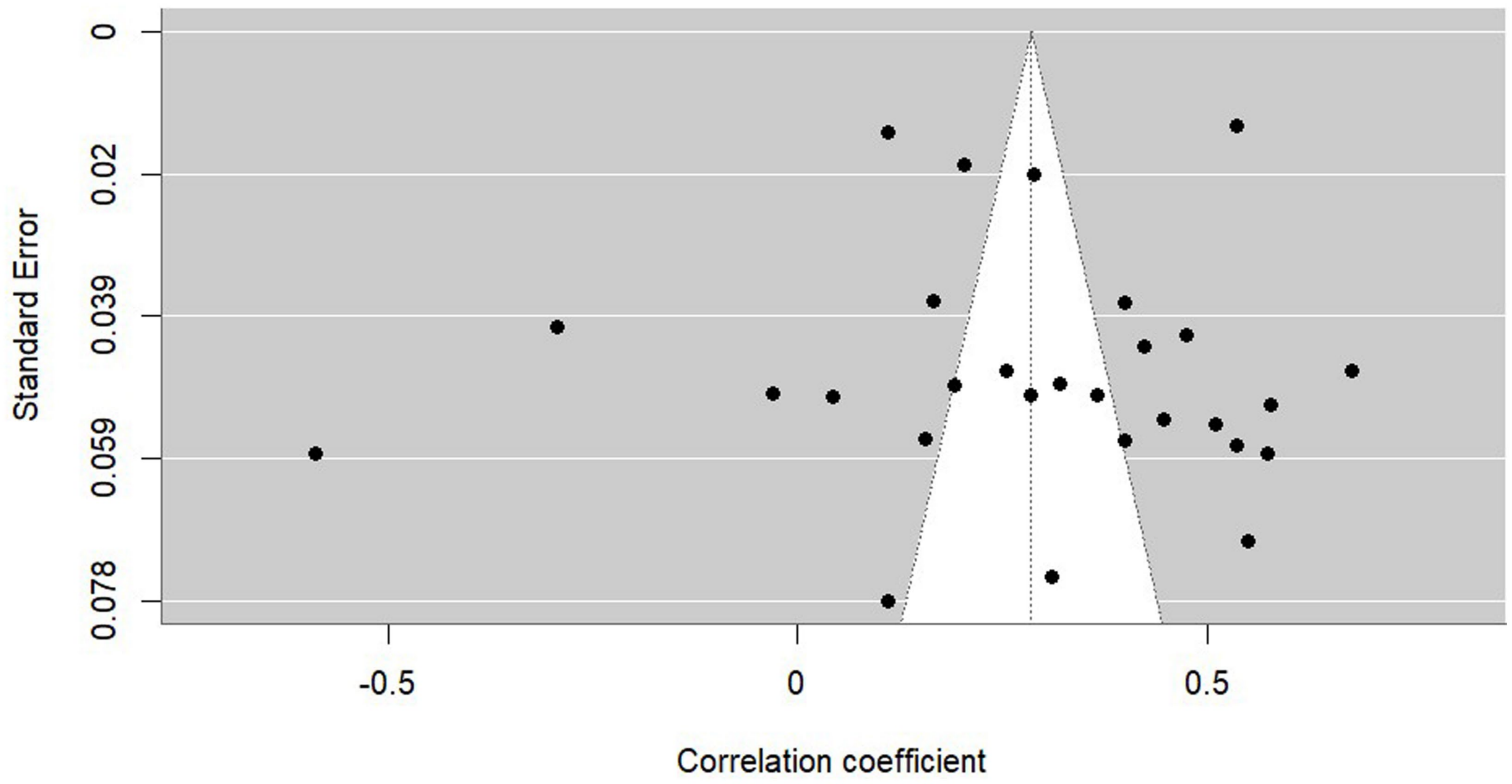
Funnel plot diagram (anxiety).

**Figure 4:**
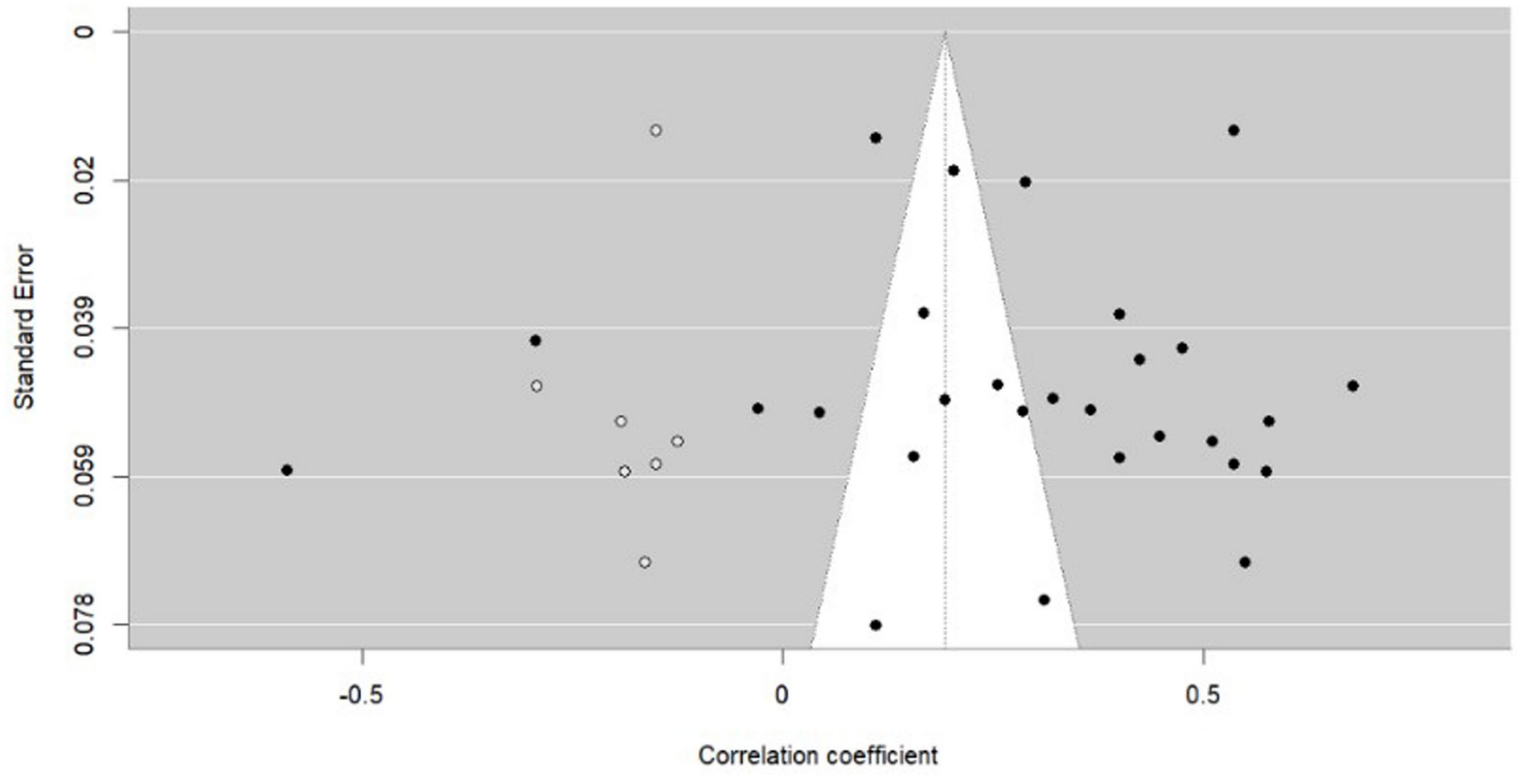
Funnel plot diagram (anxiety) after trim-and-fill Duval & Tweedie procedure.

After performing the statistical analysis to check and identify potential outliers (Bajaut diagram, diagnostics derived from the standard linear regression, leave-one-out analysis) and publication biases (funnel plot diagram—see Figure 3, Egger’s regression test, rank correlation test) with no significant influences, the Duval and Tweedie trim-and-fill procedure showed the possibility of overestimating the initial result (*r* = 0.278, 95% CI[0.182, 0.369], *p* < 0.001) due to publication bias. However, for the “real” effect, when selective publication was controlled for (see Figure 4, *r* = 0.190, 95% CI[0.089, 0.287], *p* < 0.001 rather than *r* = 0.278, 95% CI[0.182, 0.369], *p* < 0.001).^6^

##### 3.2.2.1 Moderators of the association between the general digital environment and anxiety

Considering that the heterogeneity of the studies was significant, Q_(27)_ = 1302.85, *p* < 0.001, I^2^ = 98.43%, possible moderators were also analyzed. The type of digital environment and the method of using or interacting with the digital environment (psychological concept) were analyzed as moderators of the relationship between the overall digital environment and anxiety, as shown in Table 3. The two analyzed moderators are significant; *p*-values in the case of the test of moderators was values lower than 0.05, namely 0.0361 and 0.0264, respectively.^7^

**Table 3:** Subgroup analyses of the relationship between digital environment and anxiety.

Moderator	Category	k	r	95% CI	Q	Q(df)	*p*-value
Digital environment type (digital entity)	Smartphone	17	0.328^***^	[0.270, 0.383]			
	AI	7	0.059	[0.219, 0.328]			
	SM	3	0.370^***^	[0.157, 0.550]			
	RW	1	0.109^***^	[0.082, 0.135]			
					8.5365	3	0.0361
Digital environment use (psychological concept)	Addiction	9	0.393^***^	[0.335, 0.448]			
	Problematic use	7	0.296^***^	[0.178, 0.406]			
	AI use	3	0.065	[0.378, 0.485]			
	Frequency	2	0.166^***^	[0.086, 0.243]			
	AI adoption intention	2	−0.186	[−0.695, 0.448]			
	AI user Perceptions	2	0.305^**^	[0.016, 0.547]			
	Nomophobia	1	0.379^***^	[0.305, 0.450]			
	Phubbing	1	0.300^***^	[0.212, 0.384]			
	RW experience	1	0.108^***^	[0.081, 0.135]			
					17.3787	8	0.0264

k, number of studies; ^*^p < 0.05, ^**^p ≤ 0.01, ^***^p ≤ 0.001; RW, Remote Work; AI, Artificial Intelligence; SM, Social Media.

#### 3.2.3 The association between the general digital environment and depression

The overall value of the effect size obtained using the random-effects meta-analysis model regarding the association between the general digital environment (usage) and *depression* was significant and positive (*r* = 0.275, 95% CI[0.161, 0.383], *p* < 0.001).

After performing the statistical analysis to check and identify potential outliers (Bajaut diagram, diagnostics derived from the standard linear regression, leave-one-out analysis) and publication biases (funnel plot diagram—see Figure 3, Egger’s regression test, rank correlation test) with no significant influences, the Duval and Tweedie trim-and-fill procedure showed the possibility of overestimating the initial result (*r* = 0.275, 95% CI[0.161, 0.383], *p* < 0.001) due to publication bias. However, for the “real” effect, when selective publication was controlled for, *r* = 0.212, 95% CI[0.089, 0.329], *p* < 0.001 rather than *r* = 0.275, 95% CI[0.161, 0.383], *p* < 0.001.^8^

##### 3.2.3.1 Moderators of the association between the general digital environment and depression

Considering that the heterogeneity of the study was significant, Q_(13)_ = 204.76, *p* < 0.0001, I^2^ = 96.55%, possible moderators were also analyzed. The type of digital environment, method of use, interaction with the digital environment (psychological concept), average age, and geographic region were analyzed as moderators of the relationship between the general digital environment and depression (Table 4).

**Table 4:** Subgroup analyses of the relationship between digital environment and depression.

Moderator	Category	k	r	95% CI	Q	Q(df)	*p*-value
Digital environment type (digital entity)	Smartphone	11	0.302^***^	[0.235, 0.366]			
	AI	2	−0.131^***^	[−0.202, 0.059]			
	SM	1	0.413^***^	[0.317, 0.501]			
					24.1008	2	< 0.0001
Digital environment use (psychological concept)	Addiction	8	0.347^***^	[0.306, 0.386]			
	Problematic use	3	0.193^*^	[0.036, 0.342]			
	Jobs replacement by AI	2	−0.131^***^	[−0.202, 0.059]			
	Nomophobia	1	0.327^***^	[0.249, 0.401]			
					45.4473	3	< 0.0001
Mean age					1.2421	7	0.9899
Geographical region	Asia	10	0.253^***^	[0.115, 0.381]			
	Europe	3	0.262^*^	[0.030, 0.467]			
	North America	1	0.310^**^	[0.114, 0.482]			
					0.0356	2	0.9824

k, number of studies; ^*^p < 0.05, ^**^p ≤ 0.01, ^***^p ≤ 0.001; AI, Artificial Intelligence; SM, Social Media.

As we can see in Table 4, only the moderators “the type of digital environment” and “the method of using or interacting with the digital environment” (psychological concept) are significant. Namely, the *p*-value in the case of the test of moderators, in both cases, was lower than 0.05, with values < 0.0001. Looking at the geographic region, current studies in the specialized literature have allowed comparisons between Asia (e.g., Iran, China, Singapore, Bangladesh, Tibet, South Korea), Europe (Italy, Germany), and North America (United States). Similar to the average age, this variable does not moderate the relationship between the general digital environment (digitalization) and depression.^9^

#### 3.2.4 The association between the general digital environment and stress

The overall value of the effect size obtained using the random-effects meta-analysis model regarding the association between the general digital environment (usage) and stress was significant and positive (*r* = 0.296, 95% CI[0.166, 0.417], *p* < 0.001).

After performing the statistical analysis to check and identify potential outliers (Bajaut diagram, diagnostics derived from the standard linear regression, leave-one-out analysis) and publication biases (funnel plot diagram—see Figure 3, Egger’s regression test, rank correlation test) with no significant influences, the Duval and Tweedie trim-and-fill procedure showed the possibility of overestimating the initial result (*r* = 0.296, 95% CI[0.166, 0.417], *p* < 0.001) due to publication bias. However, for the “real” effect, when selective publication was controlled for, *r* = 0.228, 95% CI[0.086, 0.360], *p* = 0.0018 rather than *r* = 0.296, 95% CI[0.166, 0.417], *p* < 0.001 (see Footnote 8).

##### 3.2.4.1 Moderators of the association between the general digital environment and stress

Considering that the heterogeneity of the study was significant, Q_(5)_ = 22.15, *p* = 0.0005, I^2^ = 90.34%, countries were analyzed as a possible moderator, as shown in Table 5. The country moderator is significant; that is, the *p*-value in the case of the test of moderators has values lower than 0.05, respectively 0.0003. Looking at the country moderator, current studies in the specialized literature have allowed comparisons only between Iran, China, Italy, and Singapore (see Footnote 9).

**Table 5:** Subgroup analyses of the relationship between digital environment and stress.

Moderator	Category	k	r	95% CI	Q	Q(df)	*p*-value
Country	Iran	2	0.294^***^	[0.262, 0.326]			
	China	2	0.335^***^	[0.214, 0.447]			
	Italy	1	−0.053	[−0.239, 0.137]			
	Singapore	1	0.462^***^	[0.219, 0.651]			
					19.02	3	0.0003

k, number of studies; ^*^p < 0.05, ^**^p ≤ 0.01, ^***^p ≤ 0.001.

This result may be explained by the fact that pandemic-related preventive measures (such as lockdowns and mobility restrictions) were implemented at different times and intensities across countries, amplifying stress differently in each context. In addition, cultural norms and coping strategies for managing digital technology use may vary across populations, potentially contributing to cross-country differences in stress responses. However, these interpretations should be treated with caution given the relatively small number of studies per country.

#### 3.2.5 The association between the general digital environment and burnout

The overall value of the effect size obtained using the random-effects meta-analysis model regarding the association between the general digital environment (usage) and *burnout* was negative and insignificant (*r* = −0.027, 95% CI[−0.418, 0.372], *p* = 0.8985). Based on the results of the statistical analysis, the digital environment (usage) was divided into two groups: smartphone, AI, and RW (see Footnote 6).

- *Meta-analysis—fixed-effects model regarding the association between the digital environment (usage) represented by the smartphone and burnout*

The overall value of the effect size obtained using the fixed-effects meta-analysis model regarding the association between the digital environment (usage), represented by smartphones, and *burnout* was significant and positive (*r* = 0.278, 95% CI[0.242, 0.314], *p* < 0.001). The fact that the results of the Q test were not significant (Q_(2)_ = 1.1175, *p* = 0.5719) and that the I^2^ value (total heterogeneity/total variability) was equal to 0% (Huedo-Medina et al., 2006; Zhang et al., 2021), led to the choice of a fixed-effects meta-analysis model. Egger’s regression test was insignificant (Egger’s intercept = 0.2411, *p* = 0.7217), indicating there was no publication bias.

- *Meta-analysis—random-effects model regarding the association between the digital environment (usage) represented by AI and RW and burnout*

The general value of the effect size obtained using the random-effects meta-analysis model regarding the association between the digital environment (usage) represented by AI and RW and *PWB* is negative and significant, *r* = −0.478, 95% CI[−0.749, 0.070], *p* = 0.0234 (in this case, the significance threshold is 0.05). Given that we analyzed only two studies, Egger’s regression test could not be applied because of the lack of statistical power and degrees of freedom.

#### 3.2.6 The association between the general digital environment and loneliness and social isolation

The overall value of the effect size obtained using the random-effects meta-analysis model regarding the association between the general digital environment (usage), loneliness, and social isolation was significant and positive (*r* = 0.191, 95% CI[0.075, 0.301], *p* < 0.001). According to the statistical analysis, the meta-analysis was highly stable (see Footnote 6).

##### 3.2.6.1 Moderators of the association between the general digital environment and loneliness and social isolation

Considering that the heterogeneity of the studies was significant, Q_(11)_ = 111.14, *p* < 0.001, I^2^ = 93.8%, possible moderators were also analyzed. Ways of using or interacting with the digital environment (psychological concept), professional activity, and geographic region were analyzed as moderators of the relationship between the general digital environment (digitalization), loneliness, and social isolation, as shown in Table 6. None of the three analyzed moderators are significant; the *p*-value in the case of the test of moderators, in all three cases, have values higher than 0.05: 0.6310, 0.5263, and 0.3559, respectively.^10^

**Table 6:** Subgroup analyses of the relationship between digital environment and loneliness and social isolation.

Moderator	Category	k	r	95% CI	Q	Q(df)	*p*-value
Digital environment use (psychological concept)	Addiction	4	0.278^***^	[0.208, 0.345]			
	Problematic use	3	0.155	[−0.251, 0.514]			
	Phubbing	2	0.209^*^	[0.036, 0.370]			
	Interaction with AI (frequency)	1	0.235^**^	[0.086, 0.374]			
	Affective commitment to the organization	1	−0.195^*^	[−0.370, 0.007]			
	Co-use	1	0.158^***^	[0.092, 0.223]			
					3.4496	5	0.6310
Professional activity	Students	6	0.232^*^	[0.043, 0.405]			
	Workers	3	0.070	[−0.182, 0.313]			
	Other activities	3	0.193^***^	[0.089, 0.293]			
					1.2838	2	0.5263
Geographical region	Asia	5	0.269^***^	[0.176, 0.357]			
	Europe	5	0.154	[−0.016, 0.315]			
	Nord America	2	0.033	[−0.488, 0.538]			
					2.0662	2	0.3559

k, number of studies; ^*^p < 0.05, ^**^p ≤ 0.01, ^***^p ≤ 0.001.

Although these moderator analyses did not yield significant results, this outcome may be theoretically explained by the fact that the negative psychosocial consequences of digitalization (e.g., loneliness and social isolation) appear to operate through similar mechanisms across different populations and contexts. In other words, whether participants were students or workers, or whether they were located in Asia, Europe, or North America, the experience of social isolation in relation to digital technology use followed comparable patterns. Another possible explanation is the relatively small number of studies available for each moderator category, which reduced statistical power and may have masked subtle differences. In addition, heterogeneity in the measurement of digital environment use (e.g., various conceptualizations of addiction or problematic use) likely further contributed to the non-significant moderation effects. Taken together, these findings suggest that the impact of digitalization on loneliness and social isolation may be relatively universal, though future research with larger and more diverse samples is needed to clarify potential context-specific effects.

#### 3.2.7 The association between the general digital environment and insomnia

The overall value of the effect size obtained using the random-effects meta-analysis model regarding the association between the general digital environment (usage) and insomnia was significant and positive (*r* = 0.217, 95% CI[0.043, 0.378], *p* = 0.0147; significance benchmark 0.05, in this case). No potential outliers or biases were identified, but the *leave-one-out* analysis revealed significant variations. After removing the study (Tang et al., 2023), the overall effect size became insignificant (*r* = 0.224, 95% CI[−0.06, 0.476], *p* = 0.1225). This indicates the need for special attention and caution in the interpretation of the results (see Footnote 6).

#### 3.2.8 The association between the general digital environment and PWB

The overall value of the effect size obtained using the random-effects meta-analysis model regarding the association between the general digital environment (usage) and *PWB* is significant and positive, *r* = 0.274, 95% CI[0.011, 0.501], *p* = 0.0412 (significance benchmark 0.05, in this case), but after running the *leave-one-out* analysis, important variations in the size of the overall effect were highlighted. When the studies were removed from the analysis, one by one (Salah et al., 2024; Chang et al., 2024; Gani et al., 2023; Chaudhuri et al., 2022), the overall effect size also became insignificant (see Footnote 6).

Inspecting the six papers included in this meta-analysis, a separation of the digital environments can be noticed. The four papers mentioned above refer strictly to the fields of AI, STT, and RW, and the other two works refer to smartphones (Ostic et al., 2021; Ejaz et al., 2023). Therefore, in this case, a meta-analysis was performed considering the divisions mentioned above.

- *Meta-analysis—the random-effects model concerning the association between the digital environment (usage) consisting of AI, STT, and RW (AISTTRW) and PWB.*

The overall value of the effect size obtained using the random-effects meta-analysis model regarding the association between the digital environment (usage) consisting of AI, STT, and RW (AISTTRW) and *PWB* was significant and positive (*r* = 0.435, 95% CI[0.238, 0.598], *p* < 0.001). According to the statistical analysis, the meta-analysis was highly stable (see Footnote 6).

- *Meta-analysis—the random-effects model regarding the association between the digital environment (use) represented by the smartphone and PWB.*

The general value of the obtained effect size, using the random-effects meta-analysis model regarding the association between the digital environment (usage) represented by the smartphone and *PWB* is insignificant and negative, *r* = −0.080, 95% CI[−0.206, 0.049], *p* = 0.2236. Given the fact that the number of studies analyzed in this case is two and the requirements of a random-effects meta-analysis model are met, namely, the result of the Q test is significant or its value I^2^ (total heterogeneity/total variability) is greater than 75% (in the given situation: I^2^ = 80.38% and Q_(1)_ = 5.0969, *p* = 0.0240), the analysis will stop at this point (without testing the fixed-effects meta-analysis model).

## 4 Discussion

The entire meta-analysis, specific to the period June 2020–June to 2024, investigated variables associated with the general digital environment (usage), seen as a whole, obtaining the widest possible picture of specialized literature. Associated variables were psychological distress, anxiety, depression, stress, burnout, loneliness, social isolation, insomnia, and PWB. From everything found in the literature, to date, this is the first meta-analysis of this type, especially with its focus on the adult population (in the specialized literature, most studies of meta-analysis type carried out on components of the current subject are performed with the target population consisting of teenagers). Adults may show different patterns compared to adolescents due to both developmental and contextual factors. For instance, adults typically have more mature cognitive and emotional regulation capacities, which can shape the way they experience and cope with digital interactions. In addition, occupational and lifestyle demands, such as work-related stress, role responsibilities, and greater exposure to RW or STT, may influence their PWB in ways that differ substantially from younger populations. Including these dimensions helps clarify why focusing on adults provides added value beyond the existing meta-analyses on teens.

The values of the associations corresponding to the whole digital environment (from the point of view of use) regarding the effect size varied from low and average values to high values (Cohen, 1988). As such, the largest positive value of the effect was obtained for the association between PWB and the use of the digital environment represented by AI, RW, and STT, *r* = 0.435. The highest negative value of the effect was obtained for the association between burnout and the use of the digital environment represented by AI and RW, *r* = −0.478. Other average effect size values obtained were represented by the association between the general digital environment (usage) and psychological distress (*r* = 0.396, distress measured in studies and *r* = 0.287, distress grouped from anxiety, depression, stress, and psychological distress measured in studies), the association between the general digital environment (use) and stress (*r* = 0.296), the association between the general digital environment (usage) and anxiety (r = 0.278), or the association between the general digital environment (use) and depression (*r* = 0.275).

Comparing the abovementioned results, as well as the full results of the meta-analysis carried out in this study (Table 2), with the results of other meta-analyses available in the literature, we can say that the tendency of the population to experience certain symptoms following intense interactions with digital technology is growing. For example, in a meta-analysis (Ivie et al., 2020) of the association between SM use and depressive symptoms in adolescents for the period 2012–2020, the calculated effect size was *r* = 0.12, 95% CI[0.04, 0.20], *p* < 0.01. Another meta-analysis (Liu et al., 2022) based on the time spent on SM and the risk of depression in adolescents (which includes studies until 09.01.2022) obtained an overall effect size equal to *r* = 0.228 (in the study, the result is expressed in OR: OR = 1.59 (95% CI [1.44, 1.77], *p* < 0.001), and was roughly converted into the Pearson correlation coefficient to be more easily compared with the results of other meta-analyses). In turn, Zhang et al. (2021) conducted a study for the period 2013–2020 to obtain a general effect size of *r* = 0.37, *p* < 0.001. Furthermore, in a meta-analysis (Ding et al., 2022) conducted for the period 2013–2021, an overall effect size regarding smartphone addiction and perceived PWB of *r* = −0.33, 95% CI[−0.37,–0.29], p < 0.001 was obtained. Taken together, these comparisons suggest both similarities and divergences between adult- and adolescent-focused findings. While effect sizes in adults are generally larger than those reported in adolescent meta-analyses (e.g., Ivie et al., 2020; Liu et al., 2022), this may reflect developmental differences such as adults’ more established cognitive and emotional regulation capacities, which may amplify the psychological impact of intensive digital engagement. In addition, contextual factors specific to adulthood—such as occupational stress, family and role responsibilities, and the integration of RW or AI technologies—introduce forms of strain less salient in adolescent populations. At the same time, certain patterns appear consistent across age groups, supporting the notion that the psychosocial consequences of digitalization are partly universal, even if their intensity and expression vary depending on developmental stage and life context.

This tendency of the population to experience certain symptoms as a result of intense interaction with digital technology actually manifests itself at a macro level, not only in relation to technological progress. This is also confirmed by official statistics. The American Psychological Association presented graphical data by age categories (American Psychological Association, 2023), and a comparison between the pre-pandemic stress level (2019) and post-pandemic (2023), where a slight upward trend can be noticed.

Although the results of the meta-analysis carried out in this study are based on a relatively small number of studies and should be treated with caution, we cannot fail to notice that they are still consistent with the reality of these days. The increasing trend in the population experiencing symptoms, especially clinical symptoms, is in fact a natural consequence of the “digital revolution or transformation” in full swing, and the process is multidisciplinary, from the economic, financial, medical, and educational fields to the artistic or creative one, actually influencing the whole society (Afonasova et al., 2019). For example, “digital transformation” and the resulting business model innovation have fundamentally changed consumer expectations and behaviors, put pressure on traditional firms, and disrupted numerous markets (Verhoef et al., 2021).

However, the increasing trend in the number of mental health problems associated with the digital environment has been discussed since 2016 (Mojtabai et al., 2016). Back then, digitalization did not enjoy the extent it has at present, a present in the course of digitalization that continues to be volatile, uncertain, complex, and ambiguous (Rodriguez and Rodriguez, 2015).

Facing an unpredictable future but also a present that is continuously changing, it is normal that the population’s level of psychological distress in relation to digitalization approaches high values (*r* = 0.396, psychological distress measured in studies). A simple literature search also showed the association between smartphones and stress, anxiety, suicide, cyberbullying, and even fake news (Bastick, 2021). However, without intervention, the vicious circle is maintained, and the symptoms increase in intensity over time. An increase in stress results in a decrease in self-control, further increasing the risk of smartphone addiction (Lei et al., 2020). We also found a reflection of this situation in the current meta-analysis; the correlation between the general digital environment (usage) and stress had a value of *r* = 0.296. As the vicious cycle continues, symptoms of depression and anxiety are often associated with poor sleep quality (Volungis et al., 2020). Therefore, the less positive consequences of digitalization are associated with insomnia, loneliness, and social isolation.

Loneliness and social isolation in an increasingly connected world should have been almost non-existent or at least not to speak of values of the general effect close to average sizes (*r* = 0.191). However, in the meta-analysis presented in this paper, insomnia obtained an average value of the association effect (*r* = 0.217), and, to a large extent, we found it explained in the literature. The need for human connection in the classic way, that is, offline and face-to-face, is making its presence felt.

Excessive smartphone use has been associated with difficulties at school or work, sleep disorders, and poor interpersonal relationships (Volungis et al., 2020). Moreover, several other studies have identified that the use of technologies, especially their excessive use, negatively effects the way we interact with others, often being associated with increased loneliness (Gomes, 2002; Reis et al., 2016; Gonçalves and dos Santos, 2022). This explains the results obtained in the current meta-analyses regarding loneliness and social isolation.

Regarding the prevalence of burnout, in the meta-analysis performed on the general digital environment (usage), an almost natural separation of results was observed between the use of digital media types (statistical explanations are mentioned in the Results section of this paper). Therefore, we discuss the measurement of the effects of the use of the digital environment represented by smartphones (*r* = 0.278) and AI and RW (−0.478). Consequently, we discuss two values of the opposite effect. While the intensive use of smartphones increases burnout, the use of AI and RW significantly decreases it. However, the recommendation for the second situation (AI and RW) is to deal with the results cautiously, at least from two points of view: the specialized literature mainly emphasizes the increase in burnout for RW, which can become a source of exhaustion in the absence of socio-emotional support (Perry et al., 2018), and the second aspect is the fact that there are only two studies analyzed. Alternatively, by simplifying a series of processes and activities, AI “takes off the employee’s shoulders” a series of intellectually “consuming” tasks, leading to decreased burnout. Nevertheless, to fully clarify this aspect, it is necessary to conduct future studies using representative samples.

However, with all the precautions mentioned above, the effect size on PWB is almost directly proportional (in a positive sense) to the decrease in burnout for the same category of digital environments (entities) used (AI, RW, STT), *r* = 0.435, reinforcing the abovementioned results regarding the concept of burnout, especially since in the analysis of PWB, the number of studies is double (four studies compared to the burnout analysis). In other words, the more we use AI and work remotely, the more PWB increases.

Returning to the effect size of the relationship between smartphone use and burnout, it is necessary to state that the heterogeneity test is not significant; the heterogeneity index has a value of 0%. The populations in the analyzed studies are very different, coming from Poland, Portugal, and China, and they have different occupations, students, and employees. This allowed the results to be extrapolated to other populations. Considering the high heterogeneity, the contributions of a series of moderators were investigated (Măricuțoiu, 2020), as seen in the previous chapter—Statistical Analysis.^11^

## 5 Limitations and directions for future research

Although this study provides important theoretical and practical information, especially in the context of today’s society, it has several limitations. The findings also suggest and open perspectives for further research.

The number of studies analyzed by variables varied from 56 (in the case of psychological distress [grouped from anxiety, depression, stress, and psychological distress]) to 2 (in the case of the burnout variable—the digital environment made up of AI and RW). Although a meta-analysis is a statistical combination of the results obtained from two or more separate studies (Deeks et al., 2019; Ramaiah and Balaji, 2023) and the conclusion of the meta-analysis is statistically more significant than the analysis of any individual research due to the increased number of subjects, greater diversity of subjects, or accumulated effects and results (Ramaiah and Balaji, 2023), we believe that in the case of analyses based on two or three studies, a larger number of studies are necessary to extract generalizable information.

In addition, the meta-analysis was based on the correlation coefficients between the use of the digital environment (general) and a series of variables such as psychological distress, anxiety, depression, stress, burnout, loneliness, social isolation, insomnia, and PWB, limiting the interpretation of the results. At the same time, most studies included in the analysis had a cross-sectional design (65 out of 77 studies), a design that does not allow any kind of chronological evaluation or analysis. Therefore, in the future, it would be useful for researchers to conduct more longitudinal analyses that measure the relationships between the use of or interaction with the digital environment and several other variables so that the dynamics of the relationships can be understood in all their complexities.

Considering that most research performed to date on the use of the digital environment has focused on smartphone technology (in the current study, 52 out of 77 studies referred to smartphones), given the current worldwide context, it is absolutely necessary that future research focus more on digital entities, such as AI and RW. We make this assertion based on statistics showing the use of AI (Generative AI) has penetrated between 21 and 46% (statistics date from the current year, 2024) in economic sectors worldwide (Statistica, 2024).

Another aspect is that future studies should include populations of all age groups when discussing adults. It is common knowledge that in the field of psychology, research is usually carried out on student samples (Sanches de Oliveira and Baggs, 2023). The studies included in this analysis are no exception to this rule, considering the average age is 30 years. Therefore, in the current case, this situation makes it difficult to generalize the results obtained in adult populations over 30 years old.

This aspect of heterogeneity should also be mentioned in the current study. In most of our research, we discussed high heterogeneity. The main reasons for a high heterogeneity can include the differences (variability) in participant characteristics, the interventions (different tools used, different methods of data collection; Măricuțoiu, 2020), and the results studied. These are called clinical heterogeneity. Variability in the study design and risk of bias is called methodological heterogeneity (Deeks et al., 2008). Heterogeneity remained high even after including the previously presented moderators in the analyses (type of digital environment, way of using or interacting with the digital environment [psychological concept], average age, geographic region, country, and professional activity). This could indicate that there may be other moderators to explain the relationships or associations analyzed; however, this opportunity remains open for future studies.

Another remark regarding future research directions includes the orientation toward studying technologies such as blockchain or the IoT, which are currently very little studied in relation to variables in the field of psychology.

Whether we want it or not, whether we accept it or not, digitalization with everything it means is already here, from smartphones to AI (in whatever form we encounter it, from ChatGPT to the reception of the hotel where we go on holiday), RW, blockchain, or the IoT. The research in the field of psychology regarding the “digital transformation” must keep up with all this technological evolution to, on the one hand, intervene in time, help the population with the necessary adapted tools, and from a psychological point of view in case it is needed and, on the other hand, for the states, through their governments, to “shape” digitalization plans according to the “response” and adaptation of the populations.

## Data Availability

Publicly available datasets were analyzed in this study. This data can be found at: all papers analyzed and included in this research can be found in Table 1 of the manuscript.

## Footnotes

^1^Additional filters related to special events, like pandemics and wars, were also used. These can be found in the online supplement.

^2^Additional information about every type of anxiety can be found in the online supplement.

^3^Additional information about the potential moderator coding can be found in the online supplement.

^4^A detailed statistical analysis related to study characteristics included in the meta-analysis can be found in the online supplement.

^5^The detailed statistical analysis can be found, together with the Forest plot diagram, in the online supplement.

^6^The detailed statistical analysis can be found in the online supplement.

^7^The detailed statistical analysis of the two moderators can be found in the online supplement.

^8^The detailed statistical analysis can be found in the online supplement, together with the Forest plot diagram.

^9^The detailed statistical analysis of the moderators can be found in the online supplement.

^10^Additional information about the professional activity moderator can be found in the online supplement.

^11^A detailed discussion about the moderator values can be found in the online supplement.
