# Supplementary figures and images for: Psychological “effects” of digital technology: a meta-analysis
Source: Front Psychol. 2025 Oct 3;16:1560516. doi: 10.3389/fpsyg.2025.1560516 (PMC12531271; doi:10.3389/fpsyg.2025.1560516)

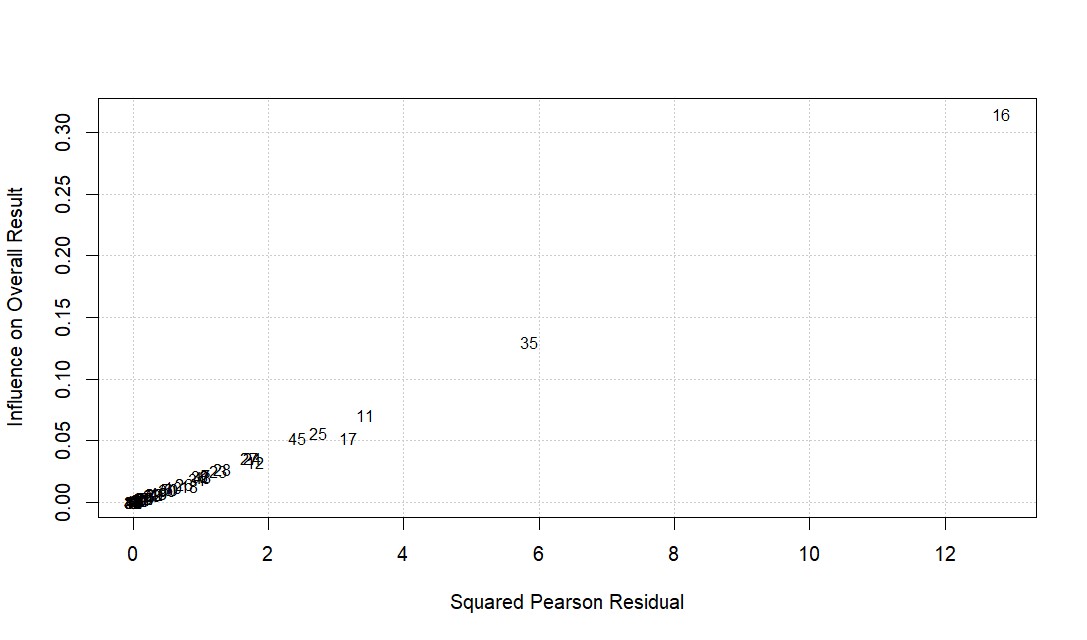

Supplement: Supplementary file 1 [file Data_Sheet_1.zip › Supplemental material figures/Figure s1.jpg]

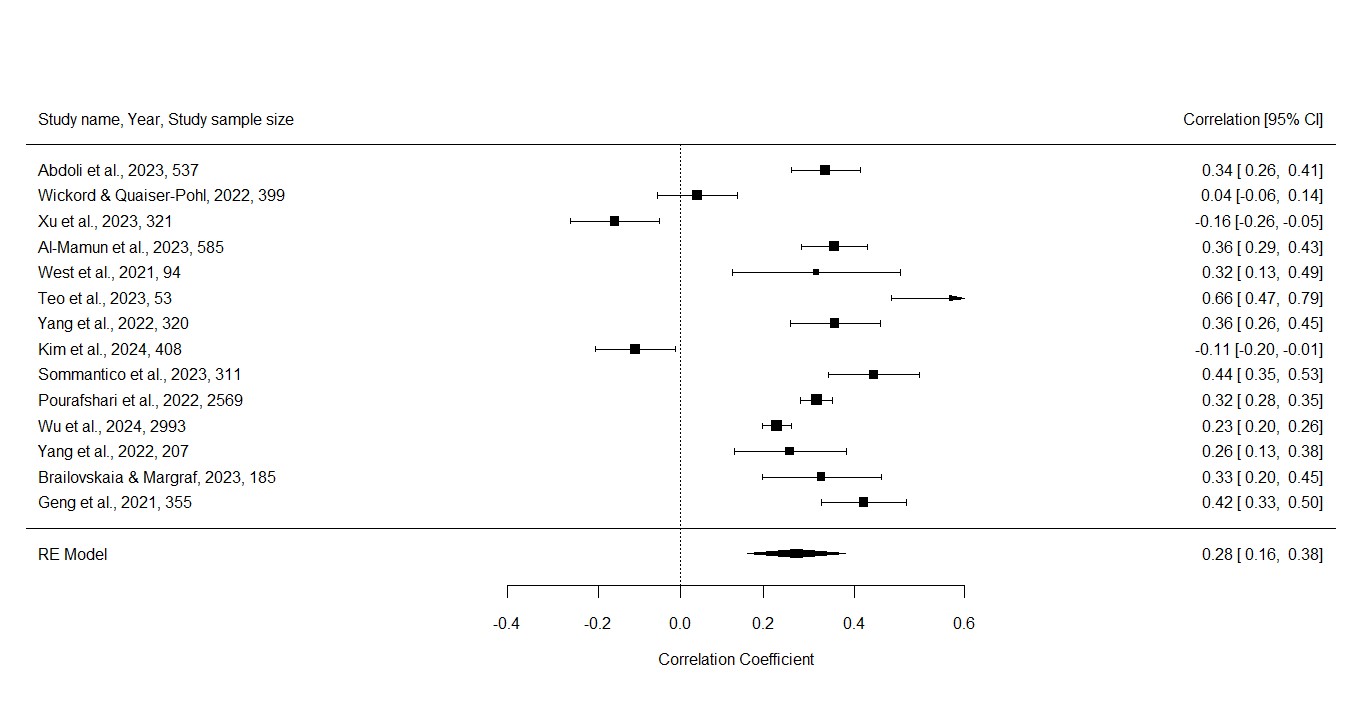

Supplement: Supplementary file 1 [file Data_Sheet_1.zip › Supplemental material figures/Figure s10.jpg]

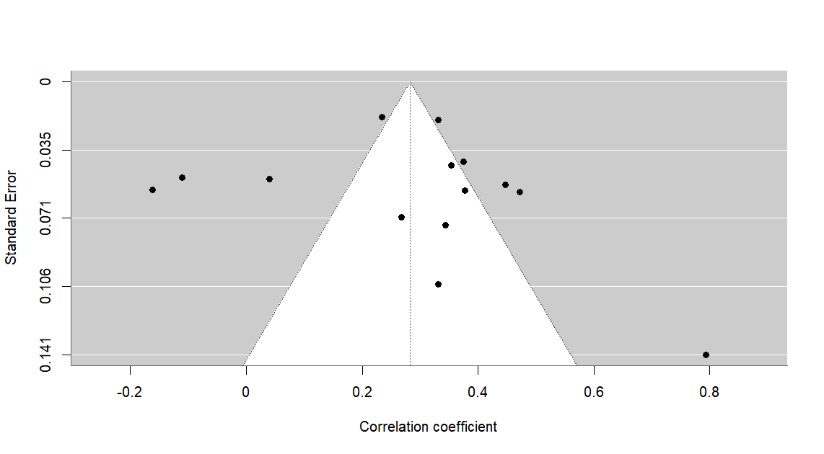

Supplement: Supplementary file 1 [file Data_Sheet_1.zip › Supplemental material figures/Figure s11.jpg]

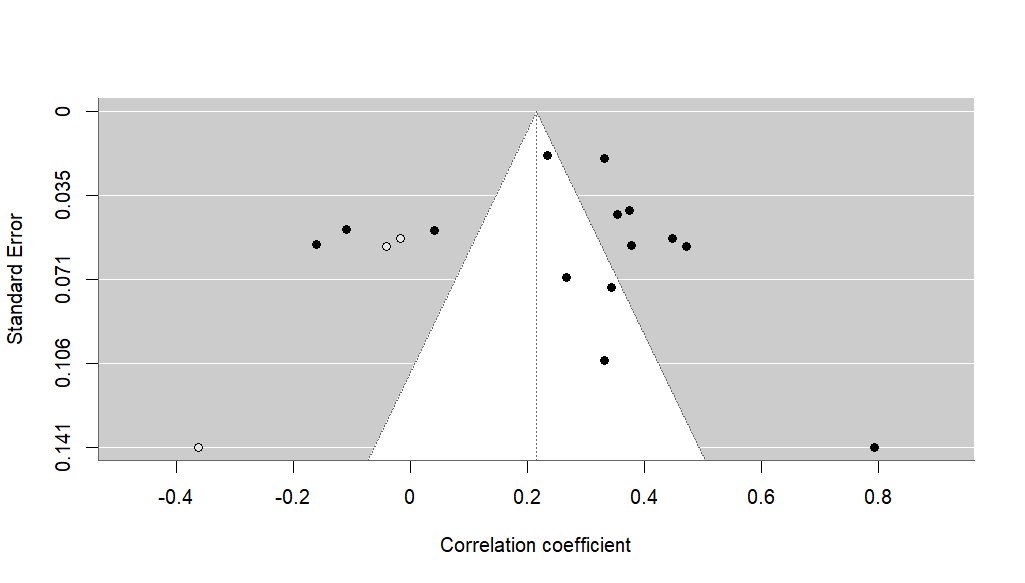

Supplement: Supplementary file 1 [file Data_Sheet_1.zip › Supplemental material figures/Figure s12.jpg]

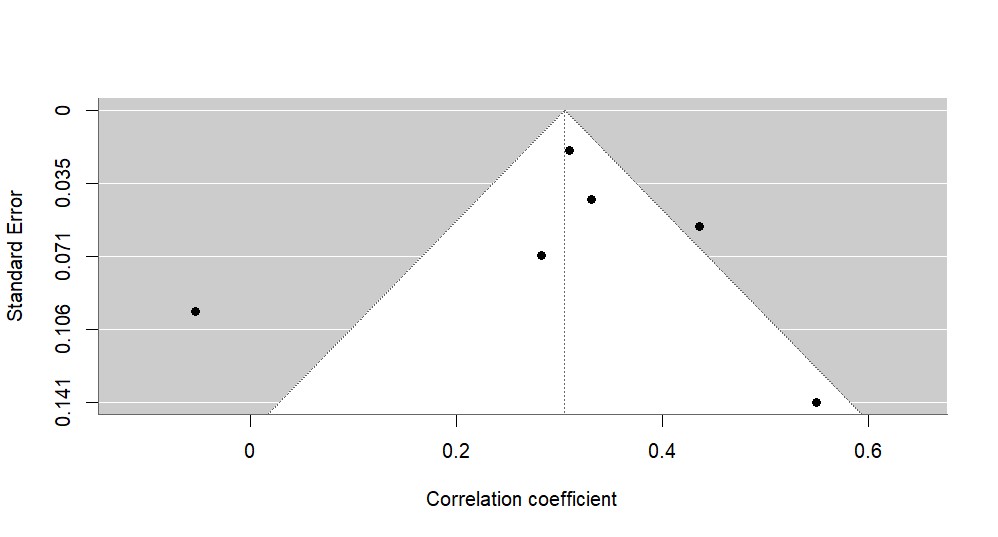

Supplement: Supplementary file 1 [file Data_Sheet_1.zip › Supplemental material figures/Figure s13.jpg]

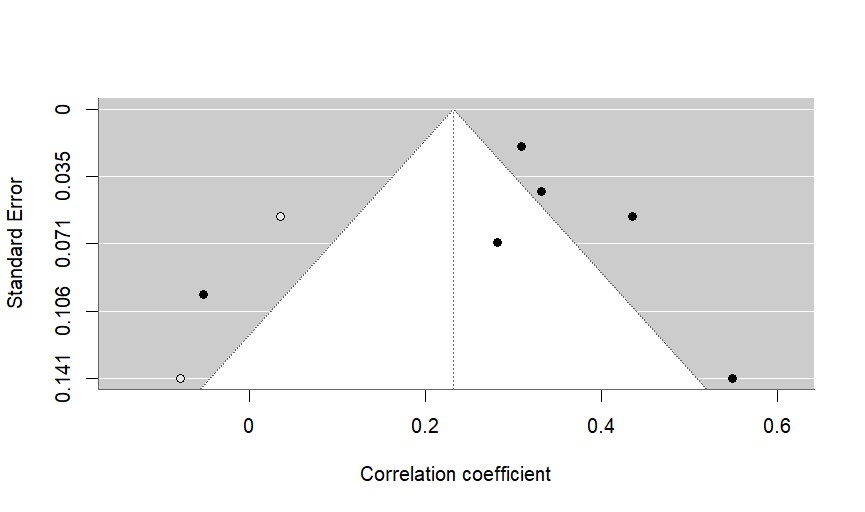

Supplement: Supplementary file 1 [file Data_Sheet_1.zip › Supplemental material figures/Figure s14.jpg]

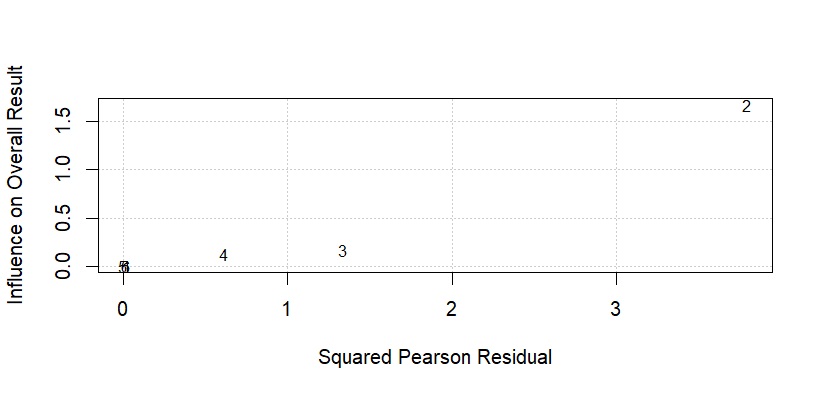

Supplement: Supplementary file 1 [file Data_Sheet_1.zip › Supplemental material figures/Figure s15.jpg]

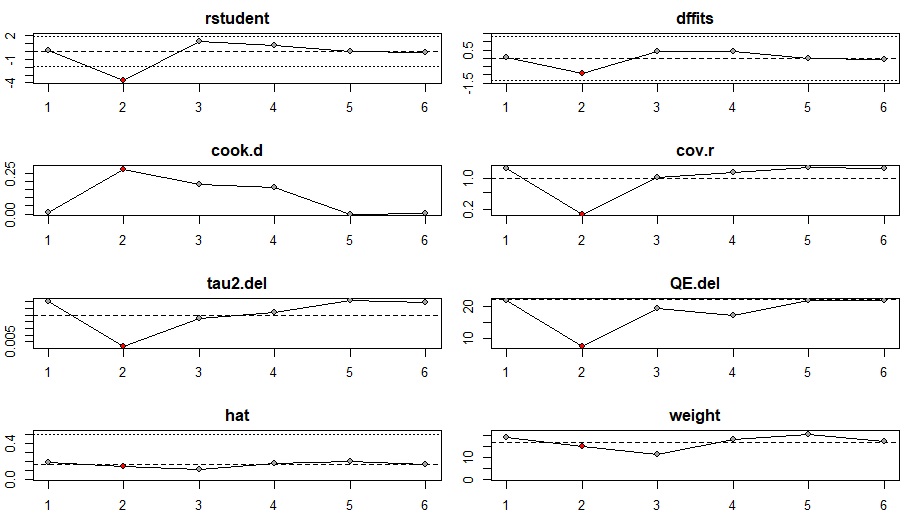

Supplement: Supplementary file 1 [file Data_Sheet_1.zip › Supplemental material figures/Figure s16.jpg]

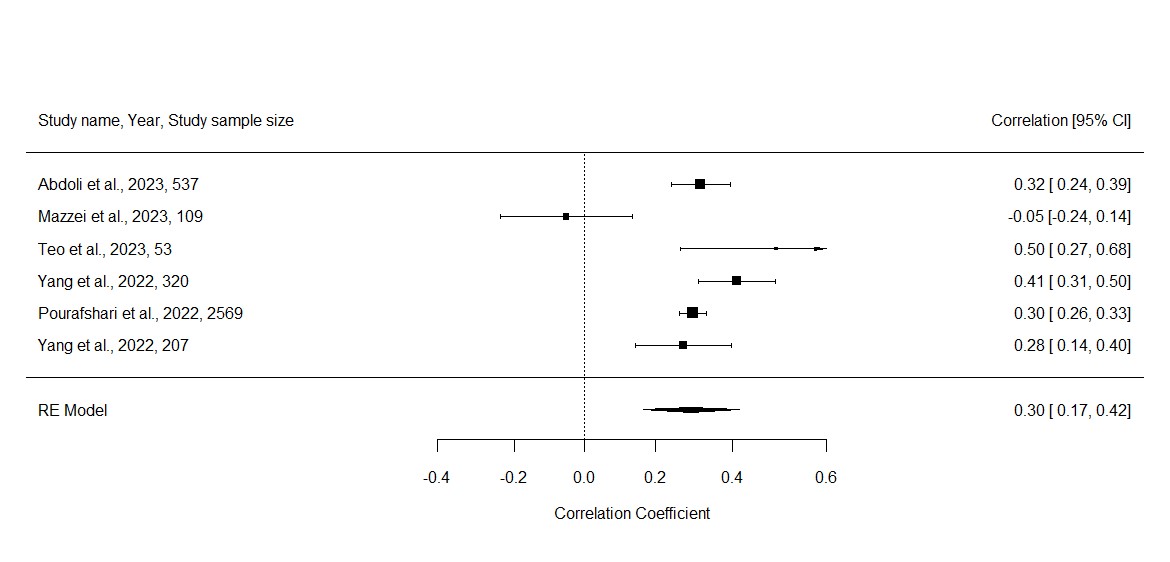

Supplement: Supplementary file 1 [file Data_Sheet_1.zip › Supplemental material figures/Figure s17.jpg]

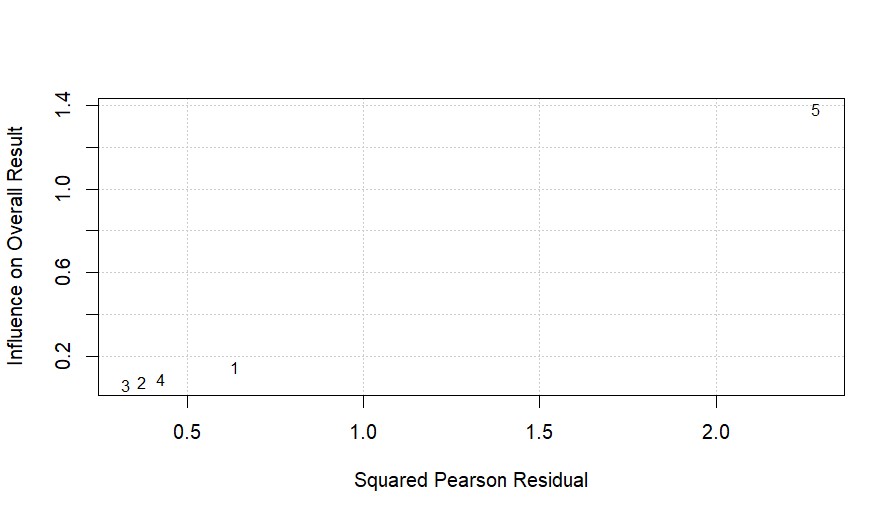

Supplement: Supplementary file 1 [file Data_Sheet_1.zip › Supplemental material figures/Figure s18.jpg]

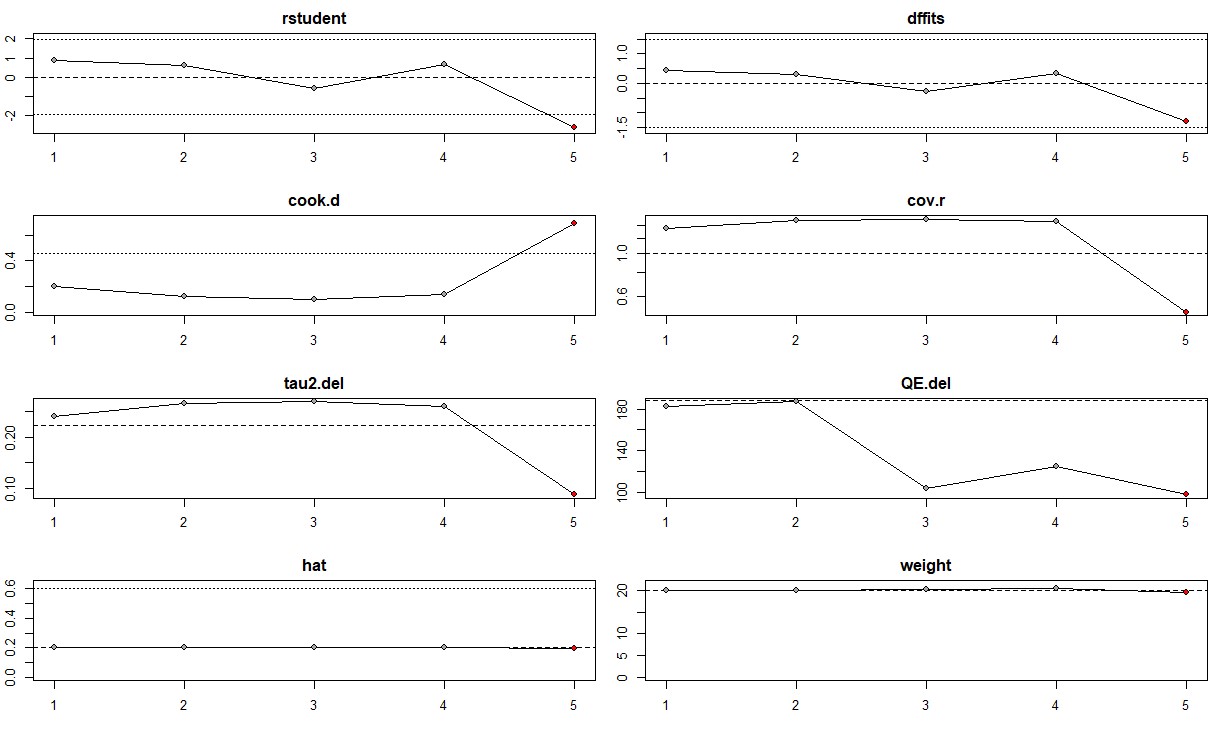

Supplement: Supplementary file 1 [file Data_Sheet_1.zip › Supplemental material figures/Figure s19.jpg]

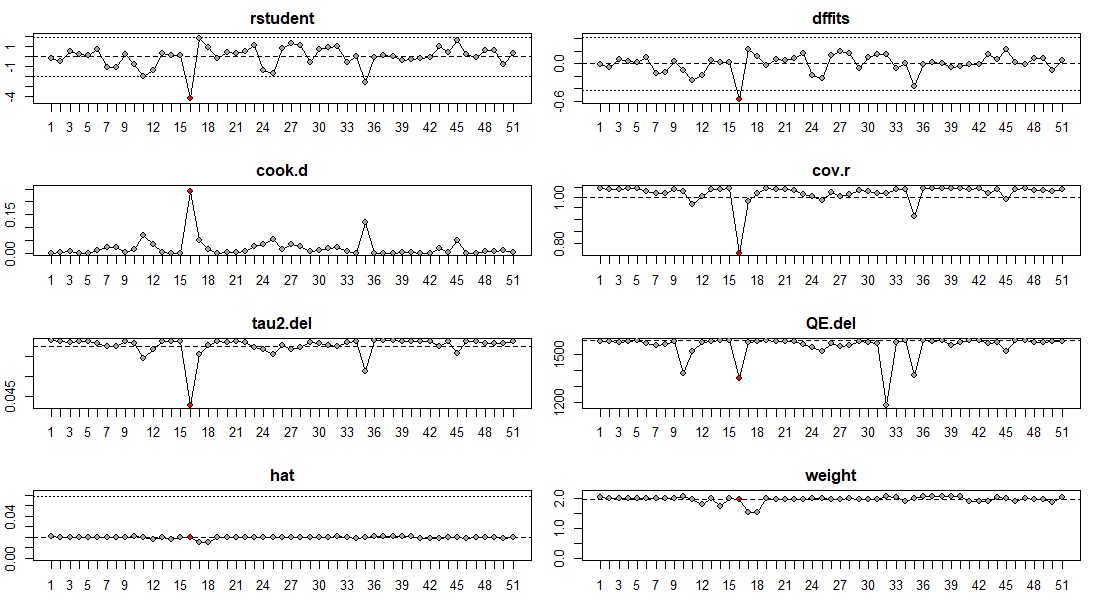

Supplement: Supplementary file 1 [file Data_Sheet_1.zip › Supplemental material figures/Figure s2.jpg]

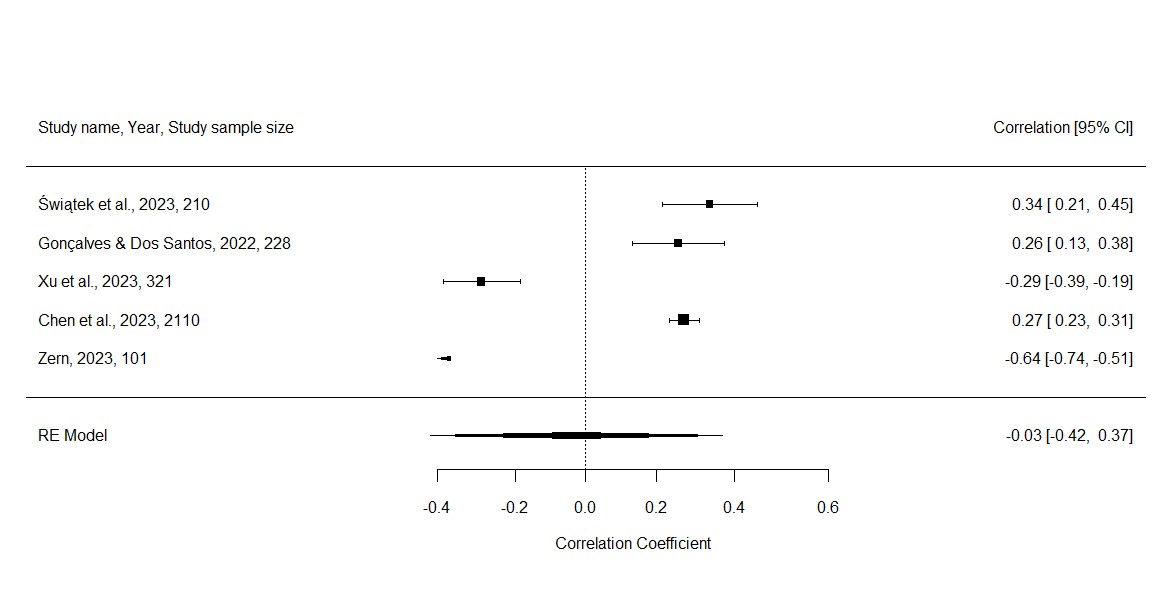

Supplement: Supplementary file 1 [file Data_Sheet_1.zip › Supplemental material figures/Figure s20.jpg]

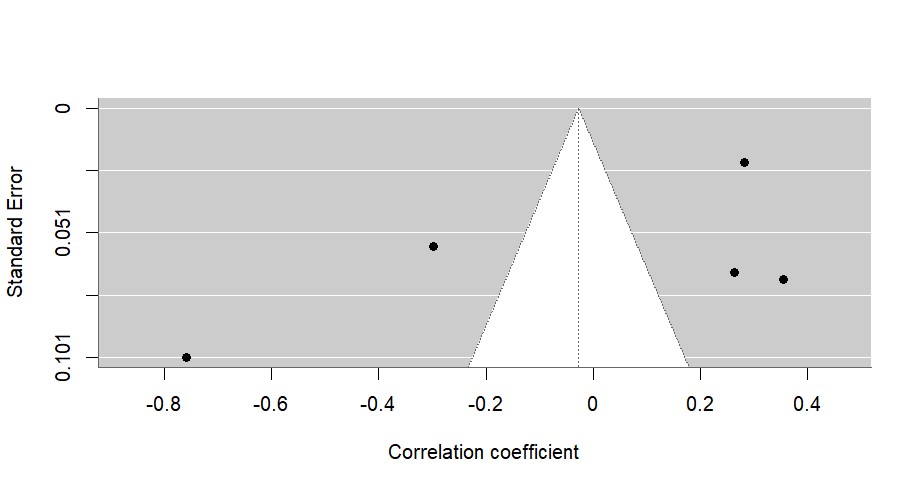

Supplement: Supplementary file 1 [file Data_Sheet_1.zip › Supplemental material figures/Figure s21.jpg]

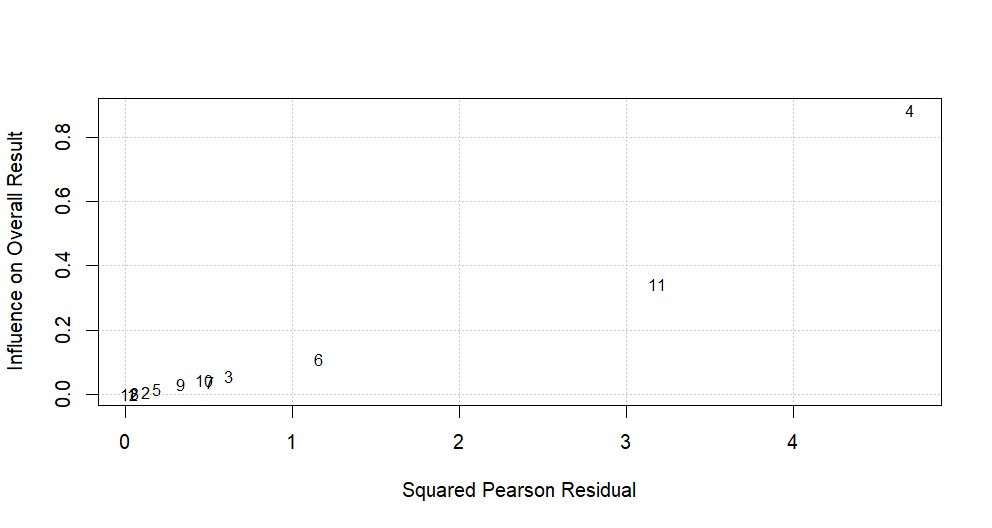

Supplement: Supplementary file 1 [file Data_Sheet_1.zip › Supplemental material figures/Figure s22.jpg]

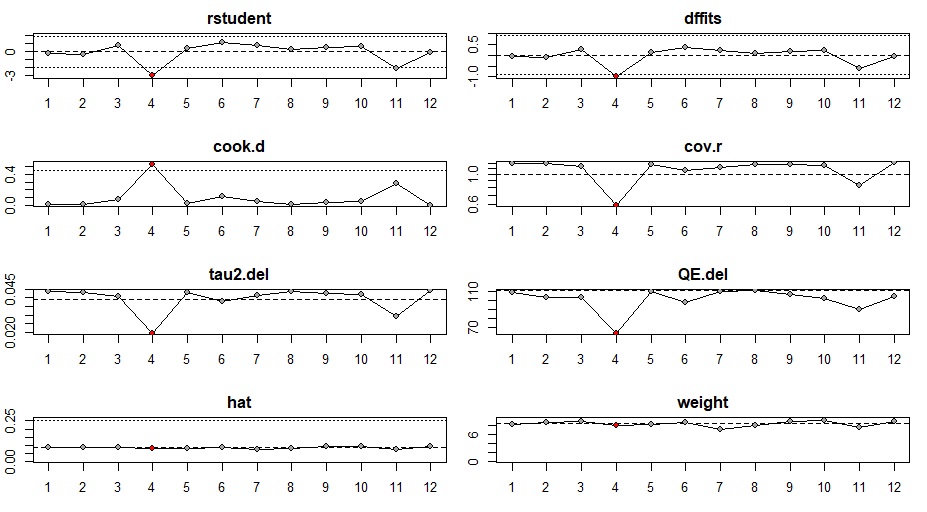

Supplement: Supplementary file 1 [file Data_Sheet_1.zip › Supplemental material figures/Figure s23.jpg]

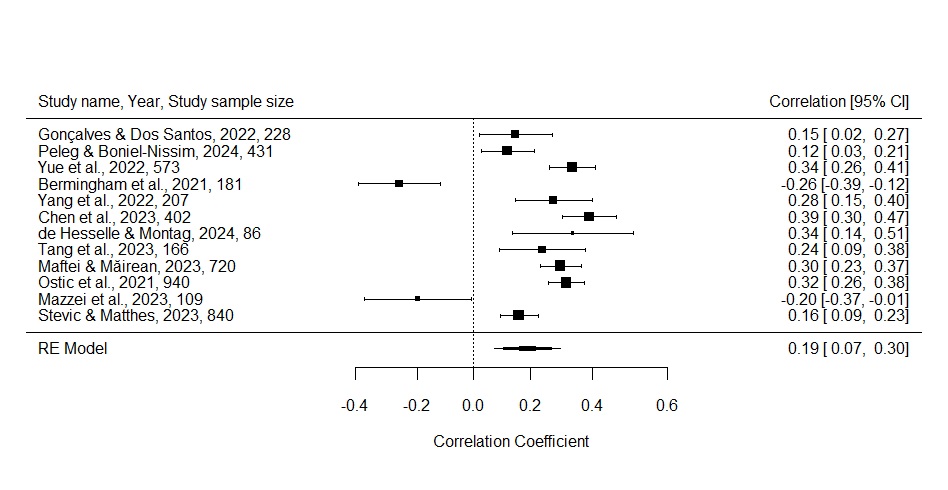

Supplement: Supplementary file 1 [file Data_Sheet_1.zip › Supplemental material figures/Figure s24.jpg]

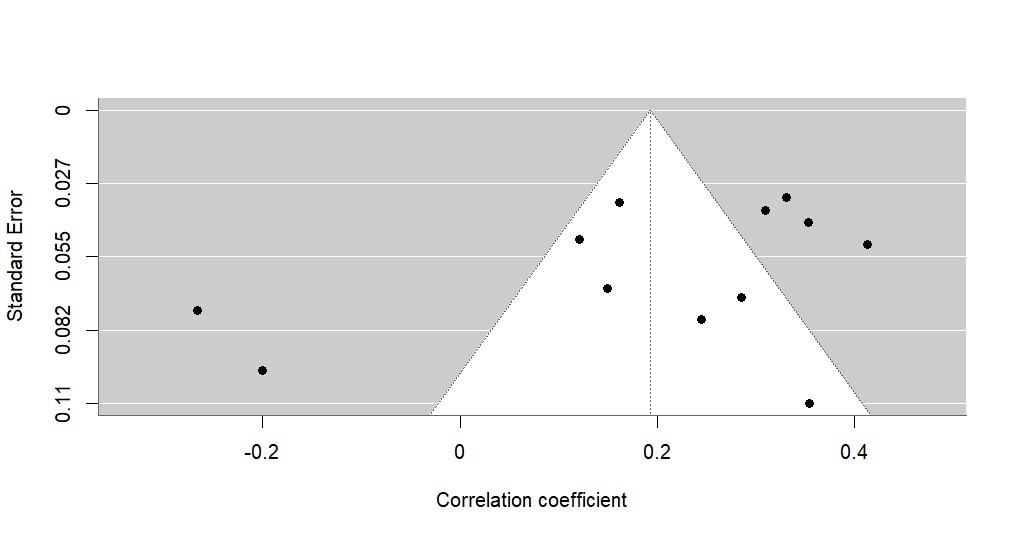

Supplement: Supplementary file 1 [file Data_Sheet_1.zip › Supplemental material figures/Figure s25.jpg]

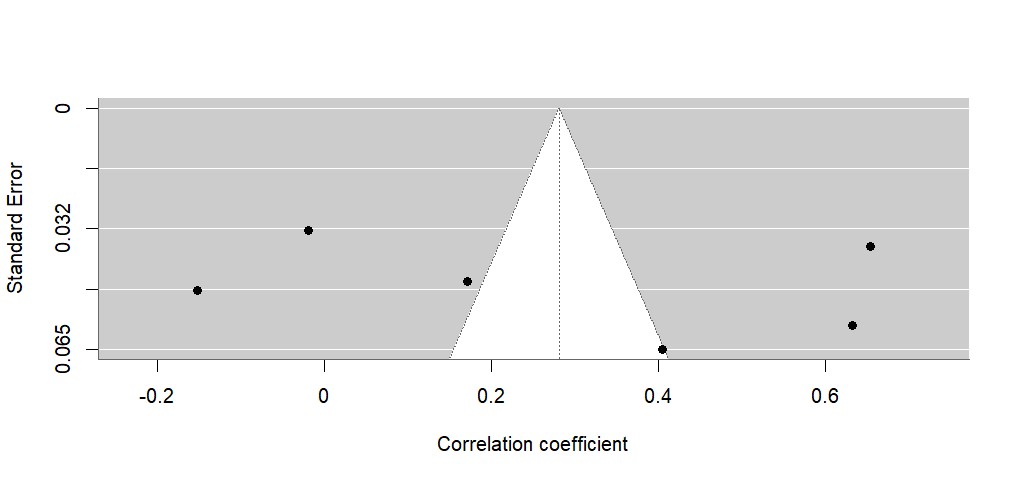

Supplement: Supplementary file 1 [file Data_Sheet_1.zip › Supplemental material figures/Figure s26.jpg]

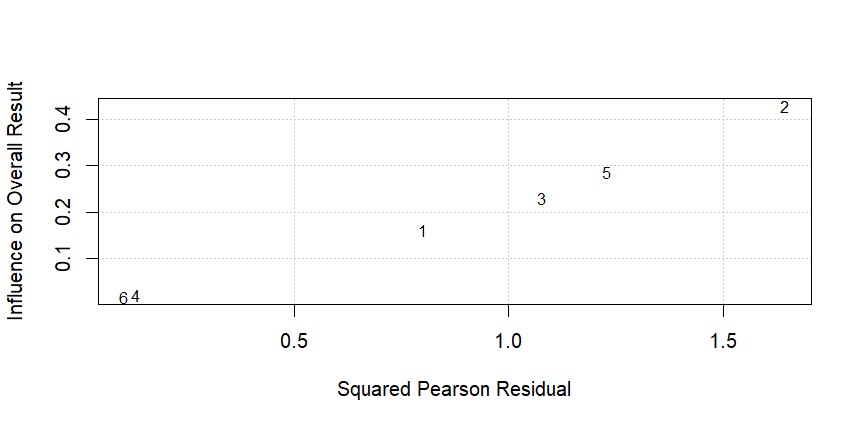

Supplement: Supplementary file 1 [file Data_Sheet_1.zip › Supplemental material figures/Figure s27.jpg]

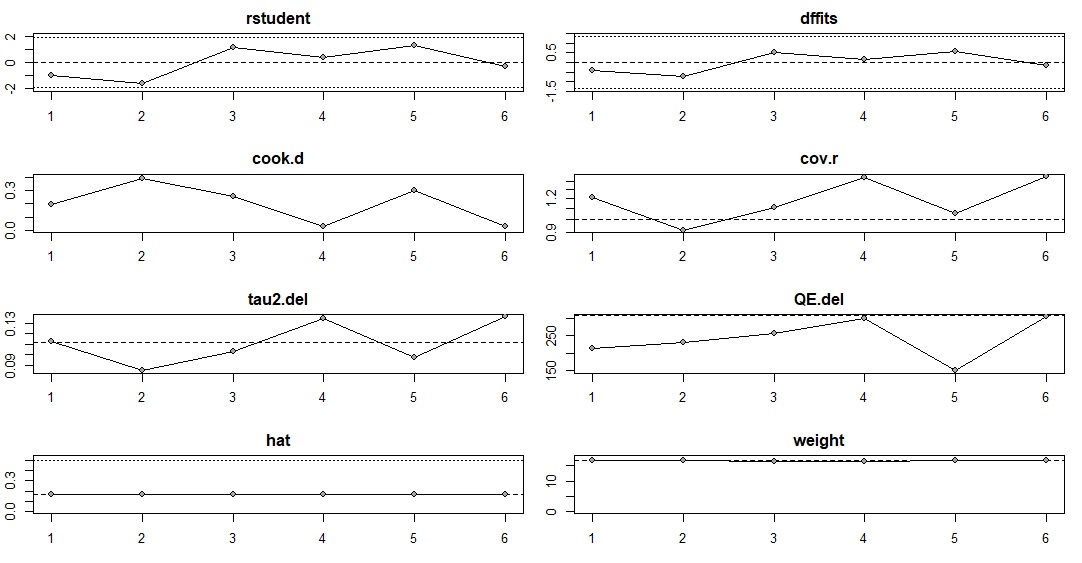

Supplement: Supplementary file 1 [file Data_Sheet_1.zip › Supplemental material figures/Figure s28.jpg]

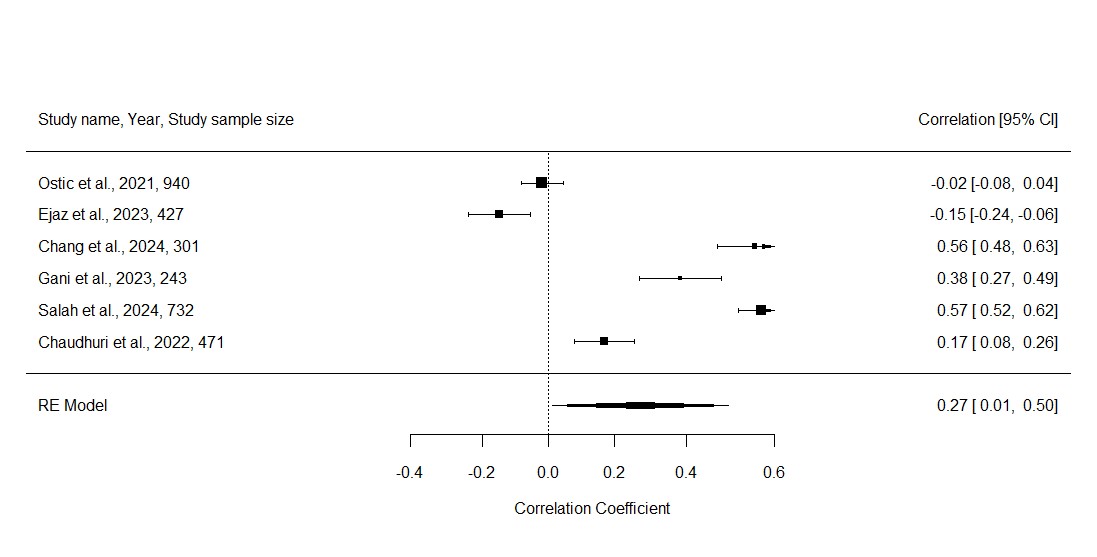

Supplement: Supplementary file 1 [file Data_Sheet_1.zip › Supplemental material figures/Figure s29.jpg]

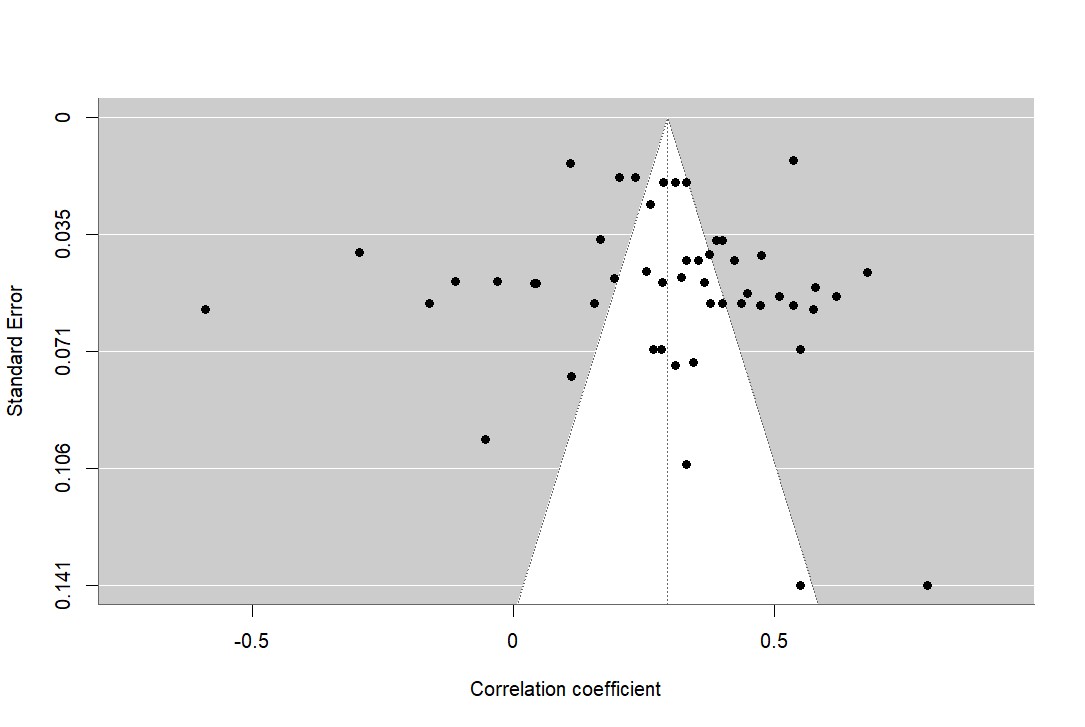

Supplement: Supplementary file 1 [file Data_Sheet_1.zip › Supplemental material figures/Figure s3.jpg]

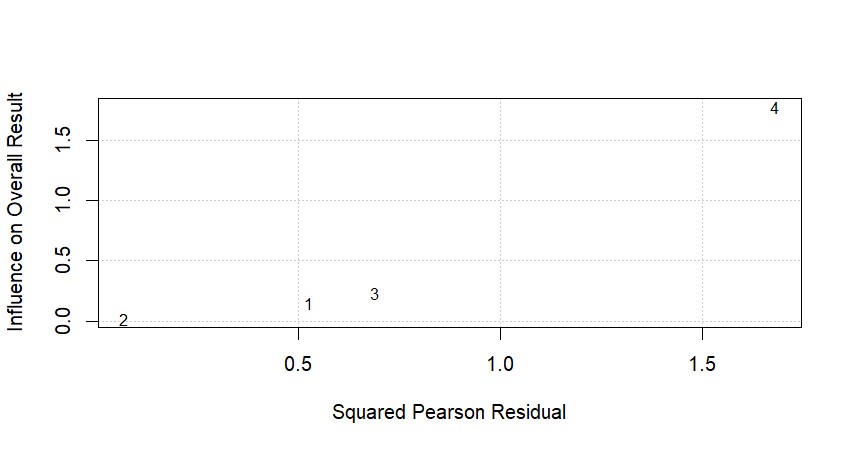

Supplement: Supplementary file 1 [file Data_Sheet_1.zip › Supplemental material figures/Figure s30.jpg]

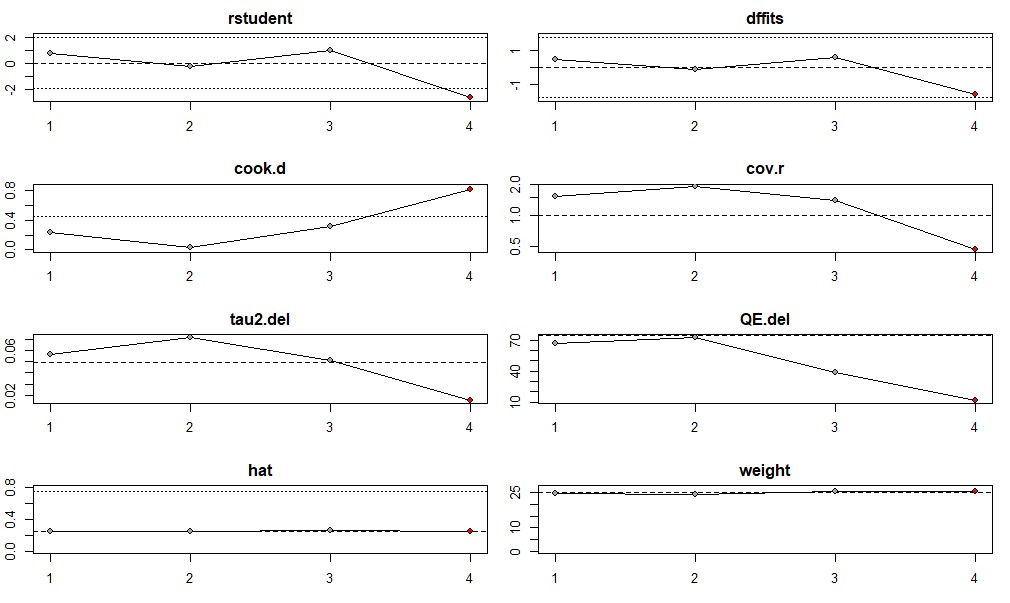

Supplement: Supplementary file 1 [file Data_Sheet_1.zip › Supplemental material figures/Figure s31.jpg]

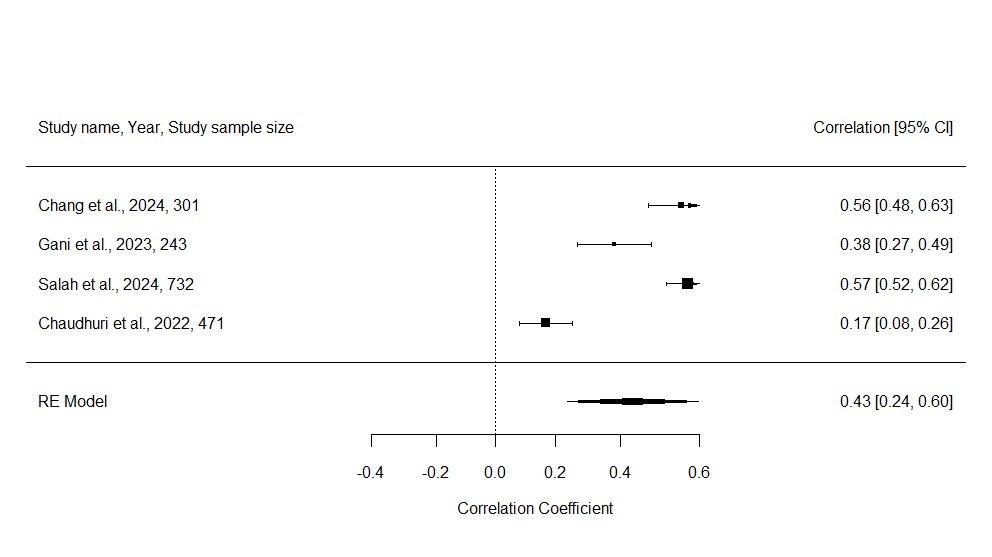

Supplement: Supplementary file 1 [file Data_Sheet_1.zip › Supplemental material figures/Figure s32.jpg]

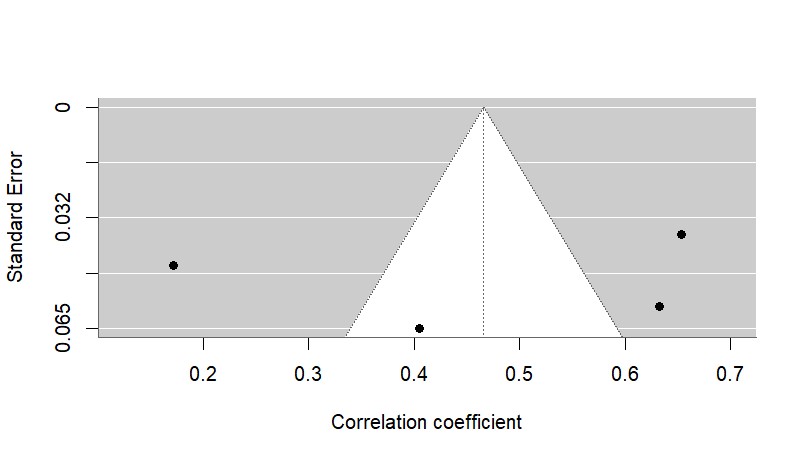

Supplement: Supplementary file 1 [file Data_Sheet_1.zip › Supplemental material figures/Figure s33.jpg]

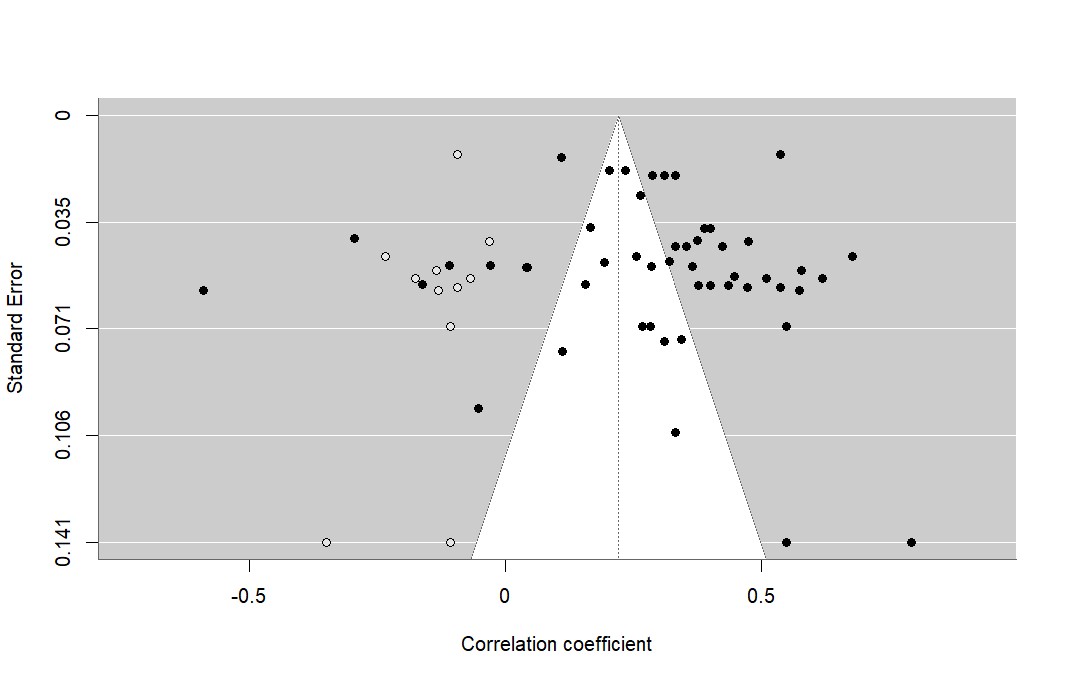

Supplement: Supplementary file 1 [file Data_Sheet_1.zip › Supplemental material figures/Figure s4.jpg]

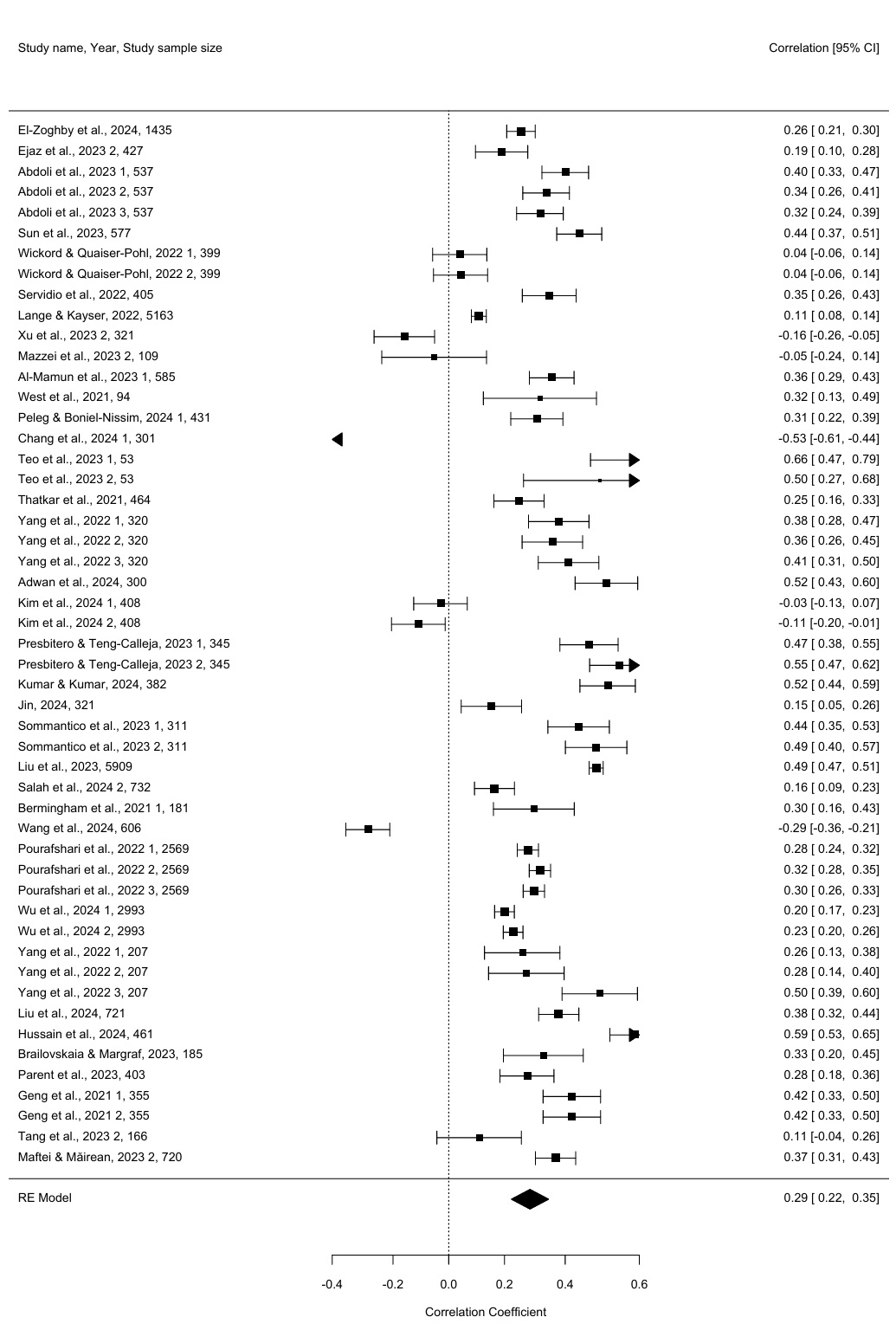

Supplement: Supplementary file 1 [file Data_Sheet_1.zip › Supplemental material figures/Figure s5.jpg]

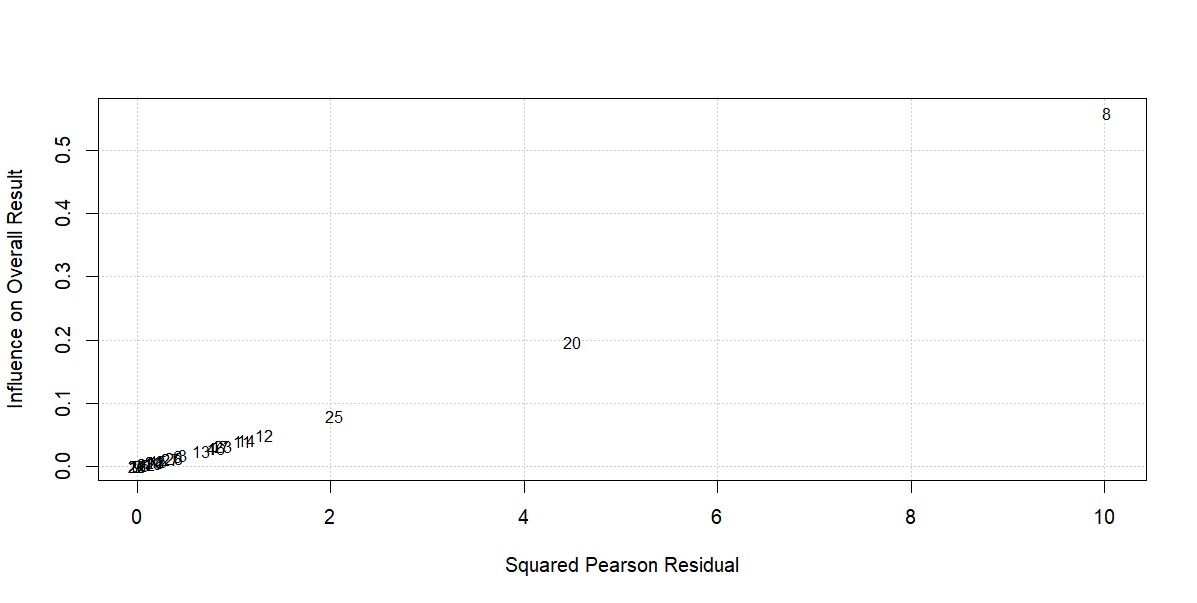

Supplement: Supplementary file 1 [file Data_Sheet_1.zip › Supplemental material figures/Figure s6.jpg]

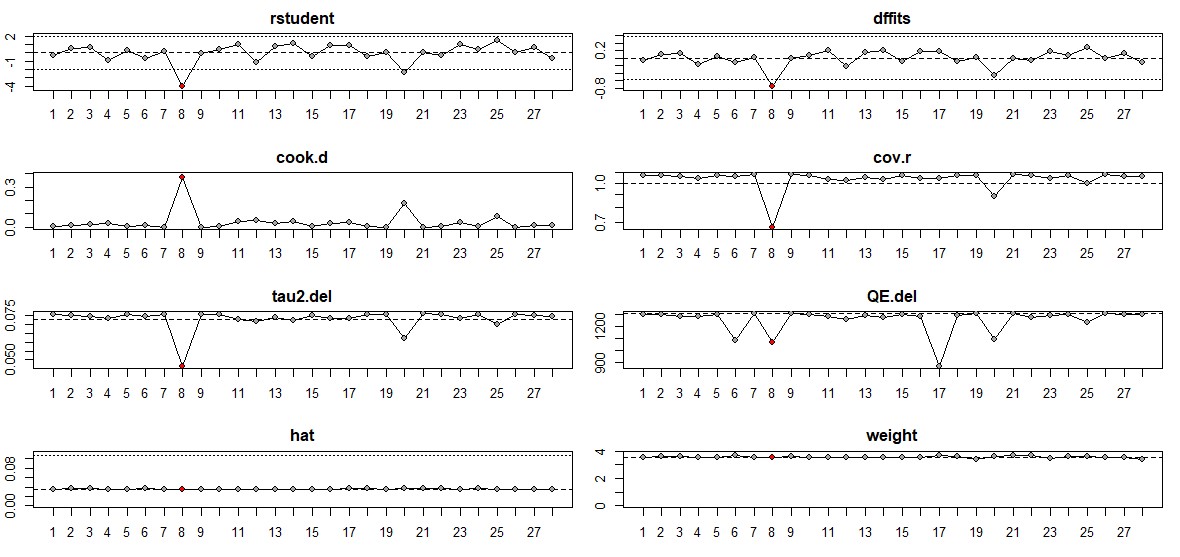

Supplement: Supplementary file 1 [file Data_Sheet_1.zip › Supplemental material figures/Figure s7.jpg]

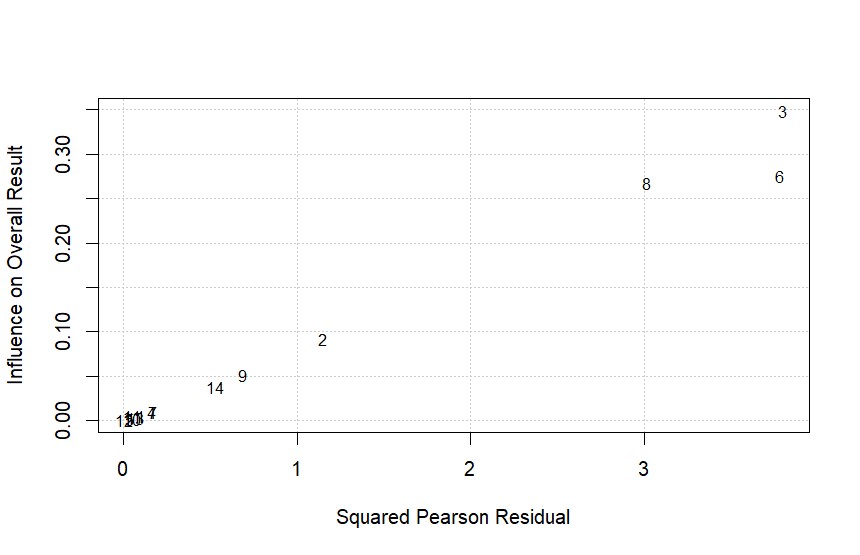

Supplement: Supplementary file 1 [file Data_Sheet_1.zip › Supplemental material figures/Figure s8.jpg]

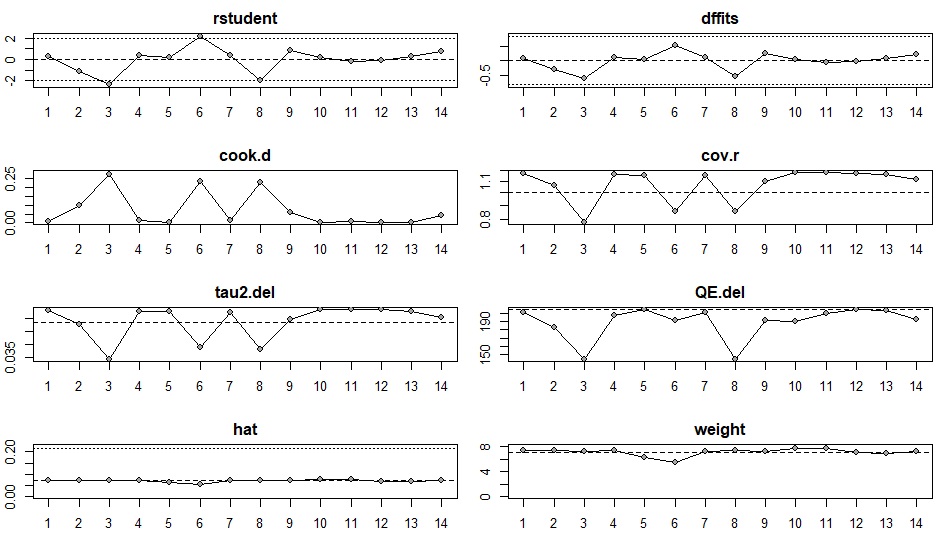

Supplement: Supplementary file 1 [file Data_Sheet_1.zip › Supplemental material figures/Figure s9.jpg]
