# Supplementary material for: Psychological “effects” of digital technology: a meta-analysis
Source: Front Psychol. 2025 Oct 3;16:1560516. doi: 10.3389/fpsyg.2025.1560516 (PMC12531271; doi:10.3389/fpsyg.2025.1560516)
Supplement: Supplementary file 2 [file Table_1.docx]

**Psychological ”Effects” of Digital Technology. Meta-analysis**

**Online Supplement**

**2.2 Inclusion and exclusion criteria – supplementary information**

Given that the purpose of this meta-analysis is to present a ”picture” as close as possible to the reality of the dynamics of the years 2020 (June) - 2024 (June) regarding the digitalization phenomenon correlated with the clinical implications at the level of mental health, and envisaging the avoidance of the potential influences generated by specific periods, such as pandemics, wars, etc., after extracting the lists of publications from the scientific databases (according to the selected search words) and analyzing each publication separately:

- the studies whose samples were generated during the periods when the pandemics occurred (for example: the period January-May 2020 - the COVID-19 pandemic - a period of high emotional intensity, which involved, in addition to the imminence of death and forced social isolation) or wars (e.g., studies conducted on the population of Ukraine starting with February 2022 – present (June 2024), considering the warfare situation);
- studies mentioning that the analyzed sample was generated before the year 2020 were removed.

We mention that the studies which did not specify the period or year of sample generation were kept, calculating the sample generation period as follows: the ”Received Date” variable of the paper by the journal minus 3 months (also, the result must exclude the period of January - May 2020). The studies which did not meet this condition were also removed. In the situation where the ”Received Date” information was not available, the calculation equation became: ”Published date/month” (or ”Accepted date/month” if the ”Published date/month” information was not available) minus 6 months, the rest of the conditions remaining unchanged. We also specify that the variable ”Ethical Approval date” (date of receipt of the Ethical Approval) regarding the conduct of the study, approval received by the authors from the institution in which the study was conducted (in the papers in which this information was mentioned) – this aspect being an additional indication, regarding the sample generation period (where the sample generation period was not mentioned in the paper).

**2.3 Data extraction and coding – supplementary information**

Supplementary information about the following subcategories which were included in the general concept of anxiety:

- FoMO (fear of missing out): FoMO is a diffuse anxiety that occurs when individuals fear that they will miss out on the positive experiences of others (referring to SM). (Huan You et al., 2018; Przybylski et al., 2013). In turn, the authors (Luca et al., 2020) define FoMO as the fear and anxiety of people who are usually connected and active on these platforms [the platforms of SM], manifested in situations where they cannot access them.
- Social phobia: In the paper (Jin, 2024), social phobia is defined as ”anxiety or apprehension (fear, restlessness) experienced in social or performance situations”.
- Job insecurity: Job insecurity refers to the perception or fear of losing one's job and includes feelings of uncertainty, anxiety and helplessness (Vander Elst et al., 2016).
- Fear of negative evaluation: Fear of negative evaluation is considered to be a hallmark of social anxiety (Clark & ​​McManus, 2002). Cognitive theories suppose that this fear may result from biased information processing, particularly when a fearful event is anticipated. In turn, the authors of the paper (Van der Molen et al., 2014) reinforce the same idea by mentioning that cognitive models assume that fear of negative evaluation is a characteristic of social anxiety.
- Social anxiety, Anxiety about working with AI, Anxiety about the professional future, global interaction Anxiety (social interaction situations), work Anxiety, attachment Anxiety.

The coding of the following *potential moderators* - supplementary information:

- type of digital environment (digital entity):
- in the category of the general concept of SM studies referring to the Metaverse subcategory were also included;
- in the category of the general concept of AI studies referring to the subcategories: ChatGPT, Generative AI, working with AI were also included;
- the remaining concepts: smartphone, RW and the technologies for smart tourism did not require coding or clustering.
- the use of the digital environment (psychological concept):
- in the category of the general concept of utilizing AI and respectively AI application, studies referring to the subcategories: AI utilizing techniques, self-efficacy in AI use, the use of Technological Pedagogical Content Knowledge in relation to the use of AI, AI application (people positions/jobs replacement by AI – at work) were also included;
- in the category of the general concept regarding AI perceptions, studies referring to the subcategories: perceived AI and ChatGPT's users perceptions were also included;
- in the category of the general concept regarding AI adoption intention, studies referring to the subcategories: Metaverse adoption for virtual learning were also included;
- in the category of the general concept of addiction, studies referring to the subcategory: overuse were also included;
- in the category of the general concept of frequency, studies referring to the subcategory: interaction with AI in terms of frequency;
- the remaining concepts: addiction, phubbing (the concept of “phubbing” refers to the way in which individuals allow distraction to permeate their interactions with partners. This involves scenarios where the romantic partner frequently interrupts communication, prioritizing prolonged cell phone use (Wang & Zhao, 2023)), problematic use, affective commitment to the organization, co-use, nomophobia (nomophobia refers to discomfort, anxiety, nervousness or anguish caused by the lack of contact with a mobile phone (Sharma et al., 2019)) did not require coding or clustering.

**2.5 Statistical Analysis – supplementary information**

The calculations were based on the z transformation (Fisher) of the correlation coefficients, r (Măricuțoiu, 2020) before the effect sizes were included in the meta-analysis, and after the calculations, for an easier interpretation, the effect size was reconverted into r (95% confidence interval for r).

I^2^ renders the degree of heterogeneity of the data used in the analysis as a percentage. However, although the Q and I^2^ Test can provide evidence for heterogeneity, they do not provide information about studies that may influence overall heterogeneity.

Egger's regression test uses regression to check the relationship between the observed effect sizes and the standard error of the observed effects. If the relationship is significant, this may indicate a publication bias. However, asymmetry can also be caused by reasons other than publication bias. (van Lissa, 2021). On the other hand, the rank correlation test evaluates whether the effect estimates and sampling variances for each study are associated. A significant test (p < 0.05) is consistent with a non-symmetric Funnel plot. However, the rank correlation test may only have moderate power for a smaller meta-analysis (Quintana, 2015).

**3.1 Characteristics of studies included in the meta-analysis - supplementary information**

The studies included in the meta-analysis addressed symptoms such as: anxiety (28 studies), burnout (5 studies), depression (14 studies), insomnia (3 studies), social isolation and loneliness (12 studies), psychological distress (3 studies) , PWB (6 studies) and stress (6 studies) in relation to the following digital environments (entities): AI (15 studies), RW(5 studies), STT (1 study), smartphone (52 studies), SM (4 studies). The most frequently measured ways of using the digital environment in the papers included in the meta-analysis were: addiction (16 studies), problematic use (11 studies) and the relationship with AI (8 studies).

We should also mention that smartphone is the ”piece of resistance” when searching for information about the digital environment in the specialized literature, in the sense that it is the entity most often studied, followed by AI, RW and SM. The classification is based on the study carried out in collecting the data for this paper and is based on the selection rules mentioned in the Methodology chapter.

**3.2 The association between digital environment (use) and other variables – supplementary information**

1. *The association between general digital environment and psychological distress*

- *The psychological distress as a grouping of the concepts of anxiety, depression, stress* *and psychological distress measured in studies* – statistical analysis

Since the heterogeneity has high values, Q_(50)_= 1581.73, p < 0.0001, I^2^= 97.84%, the Bajaut diagram (see Figure s1) and a set of diagnostics (see Figure s2) derived from the standard linear regression available in the metaphor package were constructed and inspected in turn in order to check and identify potential outliers that may influence heterogeneity. The plots highlighted that the study (Chang et al., 2024 1) had the potential to influence the analysis, but running the *leave-one out* analysis did not reveal significant variations in the overall effect size when the studies were removed from the analysis, one by one. The lowest effect value was r= 0.280, 95% CI [0.216, 0.340], p < 0.001 after removing the study (Hussain et al., 2024) and the highest value was r = 0.302, 95% CI [0.248, 0.355], p < 0.001 after removing the study (Chang et al., 2024), so it can be concluded that the results from *the leave-one* *out* analysis showed that the stability of the meta-analysis results is high. The publication biases were assessed as follows: by visual inspection of the Funnel plots (see Figure s3) and by performing tests: Egger's regression test and rank correlation test. Egger's regression test was not significant (Egger's intercept = 0.2214, p=0.3110), and the rank correlation test was also insignificant (p = 0.4400). Although the result of Egger’s regression test and the rank correlation test did not reveal the existence of a publication bias, the Duval and Tweedie trim-and-fill procedure was also run, the result of which revealed the lack of 11 studies (Estimated number of missing studies on the left side: 11 (SE = 4.7125) – see Figure s4). After redoing the meta-analysis with the potential missing studies (11), a correlation coefficient was obtained, r = 0.217, 95% CI [0.150, 0.283], p < 0.001. We can infer from this the possibility of having overestimated the initial result (r=0.287, 95% CI [0.224, 0.348], p < 0.001) due to a publication bias, however, and the ”real” effect , when selective publication is controlled to be r = 0.217, 95% CI [0.150, 0.283], p < 0.001 rather than r=0.287, 95% CI [0.224, 0.348], p < 0.001.

1. *The association between general digital environment and anxiety -* statistical analysis

Since the heterogeneity has high values, Q_(27)_= 1302.85, p < 0.0001, I^2^= 98.43%, the Bajaut plot (see Figure s6) and a set of diagnostics derived from the standard linear regression available in the metaphor package were constructed and inspected in turn (see Figure s7), in order to check and identify potential outliers that may influence it. The plots highlighted the fact that the study (Chang et al., 2024) has the potential to influence the meta-analysis, but after running the *leave-one out* analysis no important variations in the size of the overall effect were highlighted when the studies were removed from the analysis, one by one. The lowest effect value was r= 0.264, 95% CI [0.167, 0.356], p < 0.001 after removing the study (Hussain et al., 2024) and the highest value was r =0.307, 95% CI [0.229, 0.380], p < 0.001 after removing the study (Chang et al., 2024 1). All results from the *leave-one out* analysis showed that the stability of the meta-analysis results is high.

The publication biases were assessed as follows: by visual inspection of the Funnel plots (see also Figure 3) and by performing tests: Egger's regression test and rank correlation test. Egger's regression test was not significant (Egger's intercept = 0.2760, p=0.9518), and the rank correlation test was also insignificant (p = 0.4928). Although the result of Egger’s regression test and the rank correlation test did not reveal the existence of a publication bias, the Duval and Tweedie trim-and-fill procedure was also run, the result of which revealed 7 studies missing. (Estimated number of missing studies on the left side: 7 (SE = 3.5021)).

After redoing the meta-analysis with the potential missing studies (7), a correlation coefficient was obtained, r = 0.190, 95% CI [0.089, 0.287], p < 0.001. We can infer from this the possibility of having overestimated the initial result (r=0.278, 95% CI [0.182, 0.369], p < 0.001) due to a publication bias, however, and the ”real” effect , when selective publication is controlled to be r = 0.190, 95% CI [0.089, 0.287], p < 0.001 rather than r=0.278, 95% CI [0.182, 0.369], p < 0.001.

*Moderators of the association between general digital environment and anxiety*

Given the great heterogeneity between the studies, with an exploratory purpose, we divided the moderators into categories, as follows:

- the moderator *Type of digital environment (digital entity)* was divided into: Smartphone, AI, SM, RW, that is a division that moderated the results (Q_(3)_ = 8.5365, p = 0.0361). We find positive and significant effect (i.e. there is a significant relationship with anxiety) in the case of smartphone, SM and RW. Given the fact that RW totals only one study, the categories: smartphone (r = 0.328, 95% CI [0.270, 0.383], p < 0.001) and SM (r = 0.370, 95% CI [0.157, 0.550], p < 0.001) are representative for moderating the relationship. In its turn, the AI category has a positive and insignificant effect size (r = 0.059, 95% CI [0.219, 0.328], p = 0.6806).
- the moderator *Method of use or the interaction with the digital environment (psychological concept)* was divided into: Addiction, Problematic use, Use of AI, Frequency, Intention to adopt AI, User’s perceptions of AI, Nomophobia, Phubbing, The experience of working remotely, that is, a division that moderated the results (Q_(8)_=17.3787, p=0.0264). Positive and significant effect (i.e. there is a significant relationship with anxiety) we find in the case of the categories: Addiction, Problematic use, Frequency, Perceptions of users towards AI, Nomophobia, Phubbing, Experience of working remotely. Considering the fact that Nomophobia, Phubbing and Experience of RW, each add up to a single study, the categories which remain representative for moderating the relationship are: Addiction (r = 0.393, 95% CI [0.335, 0.448], p < 0.001), Problematic use (r = 0.296, 95% CI [0.178, 0.406], p < 0.001), Frequency (r = 0.166, 95% CI [0.086, 0.243], p < 0.001), users' perceptions of AI (r = 0.305, 95% CI [0.016, 0.547], p < 0.01). The Use of AI category has a positive and non-significant effect size (r = 0.065, 95% CI [0.378, 0.485], p = 0.7806) and the Intention to Adopt AI category has a negative and non-significant effect size (r = -0.186, 95% CI [-0.695, 0.448], p = 0.5828).

1. *The association between the general digital environment and depression* - statistical analysis

Since the heterogeneity has high values, Q_(13)_= 204.76, p < 0.0001, I^2^= 96.55%, the Bajaut plot (see Figure s8) and a set of diagnostics derived from the standard linear regression available in the metaphor package were constructed and inspected in turn (see Figure s9) in order to check and identify the potential outliers that may influence it. The Bajaut plot highlighted that studies (Xu et al., 2023), (Teo et al., 2023) and (Kim et al., 2024) have the potential to influence the meta-analysis, while the set of diagnoses derived from the standard linear regression did not highlight any study (no study is marked in red on any of the diagnostics). After running *the leave-one out* analysis no significant variations in the size of the overall effect were revealed when the studies were removed from the analysis, one by one. The lowest effect value was r= 0.247, 95% CI [0.140, 0.348], p < 0.001 after removing the study (Teo et al., 2023) and the highest value was r=0.304, 95% CI [ 0.205, 0.397], p < 0.001 after removing the study (Xu et al., 2023). All the results from the *leave-one out* analysis showed that the stability of the meta-analysis results is high.

The publication biases were assessed as follows: by visual inspection of the Funnel plots (see also Figure s11) and by performing tests: Egger's regression test and rank correlation test. Egger's regression test was not significant (Egger's intercept = 0.0971, p=0.1185), and the rank correlation test was also insignificant (p = 0.9145). Although the result of Egger’s regression test and the rank correlation test did not reveal the existence of a publication bias, the Duval and Tweedie trim-and-fill procedure was also run, the result of which revealed 3 studies missing (Estimated number of missing studies on the left side: 3 (SE = 2.5633)).

After redoing the meta-analysis with the potential missing studies (3) – see also Figure s12, a correlation coefficient was obtained, r = 0.212, 95% CI [0.089, 0.329], p < 0.001. We can infer from this the possibility of having overestimated the initial result (r=0.275, 95% CI [0.161, 0.383], p < 0.001) due to a publication bias, however, and the ”real” effect, when selective publication is controlled to be r = 0.212, 95% CI [0.089, 0.329], p < 0.001 rather than r=0.275, 95% CI [0.161, 0.383], p < 0.001.

*Moderators of the association between the general digital environment and depression*

Given the great heterogeneity between the studies, with an exploratory purpose, we divided the significant moderators, by category, as follows:

- the moderator Type of digital environment (digital entity) was divided into: Smartphone, AI and SM, that is a division that moderated the results (Q_(2)_ = 24.1008, p < 0.0001). We find a positive and significant effect (i.e. there is a significant relationship with depression) in the case of smartphone and SM, and we find a negative and significant effect in the case of AI. Considering the fact that the SM sum up only one study, the categories: mobile phone (r = 0.302, 95% CI [0.235, 0.366], p < 0.001) and AI (r = -0.131, 95% CI [-0.202, 0.059], p < 0.001) are representative for moderating the relationship.
- the moderator Method of use or the interaction with the digital environment (psychological concept) was divided into: Addiction, Problematic use, AI Implementation, Nomophobia, namely a division that moderated the results (Q_(3)_ = 45.4473 , p < 0.0001). We find a positive and significant effect (i.e. there is a significant relationship with depression) in the case of the categories: Addiction, Problematic use and Nomophobia. We find a negative and significant effect in the case of AI implementation (replacing workplaces with AI). Considering the fact that Nomophobia sums up only one study, the categories which remain representative for moderating the relationship are: Addiction (r = 0.347, 95% CI [0.306, 0.386], p < 0.001), Problematic use (r = 0.193, 95% CI [0.036, 0.342], p = 0.0164) and IA Implementation (r = -0.131, 95% CI [-0.202, 0.059], p < 0.01).

1. *The association between the general digital environment and stress* - statistical analysis

Since the heterogeneity has high values, Q_(5)_= 22.15, p = 0.0005, I^2^= 90.34%, the Bajaut plot (see Figure s15) and a set of diagnostics derived from the standard linear regression available in the metaphor package were constructed and inspected in turn (see Figure s16), in order to check and identify the potential outliers that may influence it. The plots highlighted the fact that the study (Mazzei et al., 2023) has the potential to influence the meta-analysis, but running *the leave-one out* analysis did not reveal important variations in the overall effect size when the studies were removed from the analysis, one by one. The lowest effect value was r= 0.268, 95% CI [0.133, 0.394], p < 0.001 after removing the study (Teo et al., 2023) and the highest value was r = 0.329, 95% CI [ 0.278, 0.378], p < 0.001 after removing the study (Mazzei et al., 2023). All the results from the *leave-one out* analysis showed that the stability of the meta-analysis results is high.

The publication biases were assessed as follows: by visual inspection of the Funnel plots (see also Figure s13) and by performing tests: Egger's regression test and rank correlation test. Egger's regression test was not significant (Egger's intercept = 0.2978, p=0.9573), the rank correlation test was also insignificant (p = 1.0000). Although the result of Egger’s regression test and the rank correlation test did not reveal the existence of a publication bias, the Duval and Tweedie trim-and-fill procedure was also run, the result of which highlighted 2 studies missing (Estimated number of missing studies on the left side: 2 (SE = 1.7439)). After redoing the meta-analysis with the potential missing studies (2) – see also Figure s14, a correlation coefficient was obtained, r = 0.228, 95% CI [0.086, 0.360], p = 0.0018. This may mean that it is possible that the initial result (r=0.296, 95% CI [0.166, 0.417], p < 0.001) was overestimated due to a publication bias, however, and the ”real” effect , when selective publication is controlled to be r = 0.228, 95% CI [0.086, 0.360], p = 0.0018 rather than r=0.296, 95% CI [0.166, 0.417], p < 0.001.

*Moderators of the association between the general digital environment and stress*

Given the great heterogeneity between the studies, with an exploratory purpose, we divided the moderator country by category, thus moderating the results (Q_(3)_ = 19.02, p = 0.0003). We find the positive and significant effect (i.e. there is a significant relationship with stress) in the case of Iran, China and the state of Singapore. Italy has a negative and non-significant effect size (r = -0.053, 95% CI [-0.239, 0.137], p = 0.5853). Considering the fact that Singapore sums up only one study, the countries representative for moderating the relationship are: Iran (r = 0.294, 95% CI [0.262, 0.326], p < 0.001) and China (r = 0.335, 95% CI [0.214, 0.447], p < 0.001).

1. *The association between the general digital environment and burnout -* statistical analysis

In its turn, not even the *leave-one out* analysis returned, through its results, the important indexes regarding the clear influence of certain studies on the overall effect size, the result obtained being: after removing the studies from the meta-analysis one by one, each time the overall size of the effect remained insignificant. Considering the given situation, the next step was to inspect the meta-analysis studies one by one but also based on the plots: Bajaut plot, a set of diagnostics derived from the standard linear regression, Forest plot and Funnel plot, which clearly highlighted two categories of trends: the first category is represented by the studies: (Xu et al., 2023) and (Zern, 2023), and the second category is represented by the studies: (Świątek et al., 2023), (Gonçalves & Santos, 2022) and (Chen^1^ et al., 2023). Analyzing each category of trends separately, we can see that the first category of studies is carried out having as digital environment (digital entity): AI and RW, and for the second category, the digital environment is represented by the smartphone, a situation that further led to the meta-analysis being divided into two categories.

1. *The association between the general digital environment and loneliness and social isolation –* statistical analysis

Since the heterogeneity has high values, Q_(11)_= 111.14, p < 0.0001, I^2^ = 93.8%, the Bajaut plot (see Figure s22) and a set of diagnostics derived from the standard linear regression available in the metaphor package were constructed and inspected in turn (see Figure s23), in order to check and identify the potential outliers that may influence it. The plots highlighted that the study (Bermingham et al., 2021) has the potential to influence the meta-analysis, but running the *leave-one out* analysis did not reveal any significant variations in the overall effect size when the studies were removed from the analysis, one by one. The lowest effect value was r= 0.170, 95% CI [0.050, 0.285], p < 0.01 after removing the study (Chen^2^ et al., 2023) and the highest value was r =0.232, 95% CI [0.144, 0.315], p < 0.001 after removing the study (Bermingham et al., 2021). All the results from the *leave-one out* analysis showed that the stability of the meta-analysis results is high.

The publication biases were assessed as follows: by visual inspection of the Funnel plots (see also Figure s25) and by performing tests: Egger's regression test and rank correlation test. Egger's regression test was not significant (Egger's intercept = 0.3913, p=0.1770), also the rank correlation test was insignificant (p = 0.3108). Although the result of Egger’s regression test and the rank correlation test did not reveal the existence of a publication bias, the Duval and Tweedie trim-and-fill procedure was also run, the result of which did not reveal any study missing (Estimated number of missing studies on the right side: 0 (SE = 1.7238)).

*Moderators of the association between the general digital environment and loneliness and social isolation*

The professional activity was extracted from the studies included in the meta-analysis and the participants were divided into three categories: students, employees and other activities (in the case of studies where the professional activity of the participants was not specified). Looking at the geographic region, the current studies in the literature allowed analyses only for Asia (Israel, China, Taiwan), Europe (Portugal, Italy, Germany, Romania) and North America (the U.S.A, Mexico).

1. *The Association between the general digital environment and insomnia* – statistical analysis

Egger's regression test was insignificant (Egger's intercept = 0.2760, p < 0.8868), and so was the rank correlation test (p = 1.0000). Although neither of these tests indicated the existence of a possible publication bias, the Duval and Tweedie trim-and-fill procedure was also run, resulting in an estimate of 0 missing studies. The next step was to perform the *leave-one-out* analysis which revealed important variations in the overall effect size when the studies were removed from the analysis, one by one: after removing the study (Tang et al., 2023), the overall effect size becomes insignificant (r=0.224, 95% CI [-0.06, 0.476], p = 0.1225), which means, first of all, special attention and caution in the interpretation of the result.

1. *The association between the general digital environment and PWB* – statistical analysis

Since the heterogeneity has high values, Q_(5)_= 306.93, p < 0.0001, I^2^ = 98.21%, the Bajaut plot (see Figure s27) and a set of diagnostics derived from the standard linear regression available in the metaphor package were constructed and inspected in turn (see Figure s28), in order to check and identify the potential outliers that may influence it. The diagrams did not highlight the studies with the potential to influence the meta-analysis, but after running the *leave-one out* analysis, important variations in the size of the overall effect were highlighted, as follows: when the studies were removed from the analysis, one by one (Salah et al., 2024), (Chang et al., 2024), (Gani et al., 2023) (Chaudhuri et al., 2022) the overall effect size also becomes insignificant.

Inspecting the 6 papers included in this meta-analysis, a separation of the digital environments can be noticed, as follows: the four papers mentioned above refer strictly to the field of AI, Intelligent Technologies for tourism and RW, and the other two works refer to the smartphone: (Ostic et al., 2021), (Ejaz et al., 2023). Therefore, the meta-analysis in this case was performed taking into account the division mentioned above.

- *Meta-analysis – the random-effects model concerning the association between the digital environment (usage) consisting of AI, Smart Technologies for tourism and RW(AISTTRW) and PWB.*

Since the heterogeneity has high values, Q_(3)_= 74.69, p < 0.0001, I^2^= 95.25%, the Bajaut plot (see Figure s30) and a set of diagnostics derived from the standard linear regression available in the metaphor package were constructed and inspected in turn (see Figure s31), in order to check and identify the potential outliers that may influence it. The plots highlighted that the study (Chaudhuri et al., 2022) has the potential to influence the meta-analysis, but running the *leave-one out* analysis did not reveal any significant variations in the overall effect size when the studies were removed from the analysis, one by one.

The lowest effect value was r= 0.381, 95% CI [0.137, 0.581], p = 0.002 after removing the study (Salah et al., 2024), and the highest value was r = 0.514, 95% CI [0.394, 0.617], p < 0.001 after removing the study (Chaudhuri et al., 2022). All the results from the *leave-one out* analysis showed that the stability of the meta-analysis results is high.

The publication biases were evaluated as follows: by visually inspecting the Funnel plots (see also Figure s33) and by performing the tests: Egger's regression test and rank correlation test. Egger's regression test was not significant (Egger's intercept = 0.5706, p=0.8759), also the rank correlation test was also insignificant (p = 0.7500). Although the result of Egger’s regression test and the rank correlation test did not reveal the existence of a publication bias, the Duval and Tweedie trim-and-fill procedure was also run, the result of which did not reveal the need to add any ”missing” study (Estimated number of missing studies on the right side: 0 (SE = 1.5649)).

**4. Discussions – supplementary information**

Positive and significant association values have been found between the general digital environment (usage) and the studied variables: psychological distress, anxiety, depression, stress, burnout (digital environment: smartphone), loneliness and social isolation, insomnia and PWB, as well as a significant negative value between burnout and the digital environment represented by AI (usage). The effect size correlation value was average in most of the results (Cohen, 1988) and, in some places, average to large. The results are consistent with what we already find in the mentioned literature; for example, in the studies (Elhai et al., 2017), (Elhai et al., 2019) and (Vahedi & Saiphoo, 2018) we find evidence that there are positive associations (the effect size being low to moderate) between problematic use of the smartphone and symptoms of depression, anxiety, stress (Squires et al., 2021).

Considering the high heterogeneity, the contributions of a series of moderators were investigated (Măricuțoiu, 2020), as follows:

- In order to analyze further the association between the *general digital environment (usage) and anxiety*, the moderators: Type of digital environment (digital entity) and Method of use or interaction with the digital environment (psychological concept) were studied.

The type of digital environment moderated the relationship between digitalization/general digital environment (usage) and anxiety. The effect size regarding the association between the general digital environment (usage) and anxiety was slightly lower in smartphone-focused users (r=0.328) than in those interested in SM (r=0.370) and insignificant in the case of AI use. The RW category, having only one case included in the analysis, shows that the result should be treated with caution.

The way of using or interacting with the digital environment (psychological concept) moderated, in its turn, the relationship between digitalization/general digital environment and anxiety. The effect size regarding the association between the general digital environment (usage) and anxiety had the highest values ​​in the case of users addicted to the digital environment (r=0.393) followed by those with problematic use behavior (r=0.296) and users' Perceptions towards AI (r=0.305). Again, AI makes a discordant note compared to the rest of the categories: Use of AI and Intention to adopt AI, the effect size being insignificant in these cases. The categories Nomophobia, Phubbing and RW Experience having only one case included in the analysis show that the results should be dealt with cautiously.

- In order to further analyze the association between the *general digital environment* *(usage) and depression*, the following moderators were studied: Type of digital environment (digital entity) and Way of using or interacting with the digital environment (psychological concept), Average Age and Geographical Region.

The type of digital environment moderated the relationship between digitalization/general digital environment (usage) and depression. The effect size regarding the association between the general digital environment (usage) and depression was average in smartphone participants (r=0.302) and negative but significant and low in AI users (r=-0.131). The category SM having included only one case in the analysis, shows that the result should be dealt with cautiously.

The way of using or interacting with the digital environment (psychological concept) also moderated the relationship between digitalization/general digital environment and depression. The effect size regarding the association between the general digital environment (usage) and depression had the highest values ​​in the case of addicted users of the digital environment (r=0.347) followed by those with problematic use behavior (r=0.193), as in the case of anxiety. As we have already stated above, AI distances itself from the other two categories by displaying a significant, low and negative effect size (Implementation of AI (replacing jobs with AI), r= -0.131). Given that we have analyzed a small number of studies, namely two, in the case of AI Implementation (replacing jobs with AI), again the results should be dealt with cautiously. The same situation in the case of Nomophobia, having included only one case in the analysis.

The Average Age and The Geographic Region did not moderate the relationship between general digital environment (usage) and depression.

In terms of Geographic Region, comparisons were made between countries in Asia (Iran, China, Bangladesh. Singapore, South Korea and Tibet), Europe (Germany, Italy) and North America (USA). However, considering that digitalization, from a technical point of view, does not take into account the country or the culture, and depression, in its turn, cannot be controlled without a psychiatric and/or therapeutic treatment plan, it is obvious why the Geographical Region cannot moderate the association general digital environment – depression.

As stated previously, the average age did not moderate the general digital environment (usage) - depression relationship either. A main reason could be the fact that, although the focus of this study was the adult population aged between 18 and 65 years, most of the age of the participants in the analyzed studies was concentrated in the range of 18-30 years, obviously the variability being, thus, small.

- In order to analyze further the association between *the general digital environment (usage) and stress*, the country moderator was studied.

The country moderated the relationship between digitalization/general digital environment and stress. A country differentiation of this moderator was also attempted, although the number of studies were reduced. So, the result was that the effect size regarding the association between the general digital environment (usage) and stress was slightly increased in the case of Chinese participants (r=0.335) and very slightly decreased in the case of Iranian participants (r=0.294). Although the two countries are totally different politically and culturally, and the number of studies in the analysis is small (two studies for each country), we cannot fail to notice that the overall effect size is very close. In other words, beyond geographical, cultural or political borders, men perceive the symptoms generated by digitalization in a similar way.

As in the previous situations, the categories with only one study in the analysis, need to be treated with caution, requiring additional studies.

- In order to analyze further the association between th*e general digital environment (usage) and loneliness and social isolation*, the moderators were studied: Way of using or interacting with the digital environment (psychological concept), Professional Activity and Geographical Region.

The Way of using or interacting with the digital environment (psychological concept), the Professional Activity and Geographical Region did not moderate the relationship between the general digital environment (usage) and loneliness and social isolation.

The nature of human contact in the era of contemporary technology has led to the decline of social relations between members of society and to the deprivation of the individual from many aspects necessary for shaping a normal personality. Therefore, technological growth and progress are seen as sources of psycho-loneliness and occasionally insecurity. (Hussien, 2022) Also, the study (MacDonald & Schermer, 2021) approaches the fact that the use of technology represented by smartphone for the person that feels lonely is not, actually, a point of interest: self-reported loneliness was related to a lower frequency of smartphone use in general and less use of communication apps. Therefore, these aspects of the literature explain why the Way of using or interacting with the digital environment is not a moderator of the general digital environment (usage) – loneliness and social isolation.

As expected, the professional activity, in its turn, does not moderate the relationship between the general digital environment (usage) and loneliness and social isolation. In fact, given the increasing trend in the percentage of RW, we can all face the problem of isolation. Issues of workplace isolation are seen as major and critical ones (Thi Thu Ha, 2021). Moreover, in the literature we even find discussions about ”digital loneliness” (Jacobs, 2024). In the paper (Walz et al., 2023) loneliness at work is also analyzed and the authors conclude that often employees simply cannot engage in social relationships because they have limited mental resources. These resources must be distributed among various tasks and relationships, and this can create interferences, especially in a challenging RW environment. Considering the current context as well as the above-mentioned things from the literature, it is clear why the professional activity cannot be a moderator of the digitalization - loneliness and social isolation.

The association between the general digital environment (usage) and loneliness is not moderated by the geographic region either. Comparisons were made between countries in Asia (Israel, China, Taiwan), Europe (Portugal, Germany, Romania, Italy) and North America (the U.S.A., Mexico). As we have already stated above, digitalization, from a technical point of view, does not take into account country or culture, and feeling lonely or isolated nowadays, although it seems that a ”whole world” can be close to you only ”within a click distance”, has to do with human nature and the need for genuine connection rather than a connection through an ”intermediary” (the digital environment). Obviously, all these feelings can be experienced beyond any geographical border.

**Additional References**

Clark, D. M., & McManus, F. (2002). Information processing in social phobia. *Biological Psychiatry*, *51*(1), 92–100. <https://doi.org/10.1016/S0006-3223(01)01296-3>

Elhai, J. D., Dvorak, R. D., Levine, J. C., & Hall, B. J. (2017). Problematic smartphone use: A conceptual overview and systematic review of relations with anxiety and depression psychopathology. *Journal of Affective Disorders*, *207*, 251–259.

<https://doi.org/10.1016/j.jad.2016.08.030>

Elhai, J. D., Levine, J. C., & Hall, B. J. (2019). The relationship between anxiety symptom severity and problematic smartphone use: A review of the literature and conceptual frameworks. *Journal of Anxiety Disorders*, *62*, 45–52.

<https://doi.org/10.1016/j.janxdis.2018.11.005>

Huan You, C., Geng Feng, N., Xiao Wei, C., Qi, W., Yu Hong, S., & Xiao Jun, S. (2018). Fear of missing out: What have I missed again? *Advances in Psychological Science*, *26*(3), 527. <https://doi.org/10.3724/SP.J.1042.2018.00527>

Hussien, R. M. (2022). The association between nomophobia and loneliness among the general population in the Kingdom of Saudi Arabia. *Middle East Current Psychiatry*, *29*(1), 68. <https://doi.org/10.1186/s43045-022-00235-8>

Jacobs, K. A. (2024). Digital loneliness—changes of social recognition through AI companions. *Frontiers in Digital Health*, *6*. <https://doi.org/10.3389/fdgth.2024.1281037>

Luca, L., Burlea, S. L., Chirosca, A.-C., Marin, I. M., Ciubara, A. B., & Ciubara, A. (2020). The FOMO Syndrome and the Perception of Personal Needs in Contemporary Society. *Brain. Broad Research in Artificial Intelligence and Neuroscience*, *11*(1Sup1), 38–46. <https://doi.org/10.18662/brain/11.1sup1/27>

MacDonald, K. B., & Schermer, J. A. (2021). Loneliness unlocked: Associations with smartphone use and personality. *Acta Psychologica*, *221*, 103454.

<https://doi.org/10.1016/j.actpsy.2021.103454>

Przybylski, A. K., Murayama, K., DeHaan, C. R., & Gladwell, V. (2013). Motivational, emotional, and behavioral correlates of fear of missing out. *Computers in Human Behavior*, *29*(4), 1841–1848. <https://doi.org/10.1016/j.chb.2013.02.014>

Sharma, M., Amandeep, Mathur, D., & Jeenger, J. (2019). Nomophobia and its relationship with depression, anxiety, and quality of life in adolescents. *Industrial Psychiatry Journal*, *28*(2), 231. <https://doi.org/10.4103/ipj.ipj_60_18>

Squires, L. R., Hollett, K. B., Hesson, J., & Harris, N. (2021). Psychological Distress, Emotion Dysregulation, and Coping Behaviour: a Theoretical Perspective of Problematic Smartphone Use. *International Journal of Mental Health and Addiction*, *19*(4), 1284–1299. <https://doi.org/10.1007/s11469-020-00224-0>

Thi Thu Ha, N. (2021). Workplace isolation in the growth trend of remote working: A Literature review. *Review of Economic and Business Studies*, *14*(1), 97–113. <https://doi.org/10.47743/rebs-2021-1-0005>

Vahedi, Z., & Saiphoo, A. (2018). The association between smartphone use, stress, and anxiety: A meta‐analytic review. *Stress and Health*, *34*(3), 347–358.

<https://doi.org/10.1002/smi.2805>

Vander Elst, T., De Cuyper, N., Baillien, E., Niesen, W., & De Witte, H. (2016). Perceived control and psychological contract breach as explanations of the relationships between job insecurity, job strain and coping reactions: Towards a theoretical integration. *Stress and Health*, 32(2), 100–116.

Van der Molen, M. J. W., Poppelaars, E. S., Van Hartingsveldt, C. T. A., Harrewijn, A., Gunther Moor, B., & Westenberg, P. M. (2014). Fear of negative evaluation modulates electrocortical and behavioral responses when anticipating social evaluative feedback. *Frontiers in Human Neuroscience*, *7*. <https://doi.org/10.3389/fnhum.2013.00936>

Walz, T., Kensbock, J. M., de Jong, S. B., & Kunze, F. (2023). Lonely@Work@Home? The impact of work/home demands and support on workplace loneliness during remote work. *European Management Journal*. <https://doi.org/10.1016/j.emj.2023.05.001>

Wang, X., & Zhao, K. (2023). Partner Phubbing and Marital Satisfaction: The Mediating Roles of Marital Interaction and Marital Conflict. *Social Science Computer Review*, *41*(4), 1126–1139. <https://doi.org/10.1177/08944393211072231>

| **Original subscale (as reported in primary studies)** | **Categorized as** |
| --- | --- |
| FoMO | Anxiety |
| Social Anxiety / Social Phobia | Anxiety |
| Job Anxiety / Job Insecurity | Anxiety |
| AI-related Anxiety (e.g., AI anxiety, professional future) | Anxiety |
| Attachment Anxiety | Anxiety |
| Fear of Negative Evaluation | Anxiety |
| Global Interaction Anxiousness | Anxiety |
| General Anxiety | Anxiety |
| Perceived Stress | Stress |
| Technostress | Stress |
| Social Media Fatigue (cognitive, behavioral, emotional) | Burnout |
| Emotional Exhaustion | Burnout |
| Learning Burnout | Burnout |
| General Exhaustion | Burnout |
| Workplace Social Isolation | Loneliness / Social Isolation |
| Capacity to Be Alone (Solitary) | Loneliness / Social Isolation |
| Loneliness (unspecified) | Loneliness / Social Isolation |
| Positive Affect (PANAS, MHI-38) | PWB |
| Tourist PWB | PWB |

Note. FoMO = Fear of Missing Out; AI = Artificial Intelligence; PWB = Psychological Well-Being; PANAS = Positive and Negative Affect Schedule; MHI-38 = Mental Health Inventory (MHI-38)

Supplementary Table S1. Categorization of anxiety, stress, burnout, loneliness, and PWB subscales used in the included studies
